# Autophagy-driven lipid regulation by an herbal decoction alleviates cardiac lipotoxicity in severe acute pancreatitis

**DOI:** 10.7150/thno.117243

**Published:** 2025-08-11

**Authors:** Yue Yang, Qian Hu, Hongxin Kang, Long Xie, Yue Hu, Juan Li, Xianlin Zhao, Lv Zhu, Wenfu Tang, Jingping Liu, Christopher J. Lyon, Meihua Wan

**Affiliations:** 1 West China Center of Excellence for Pancreatitis, Institute of Integrated Traditional Chinese and Western Medicine, West China Hospital, Sichuan University, Chengdu 610041, China.; 2 The First People's Hospital of Shuangliu District, Chengdu 610299, China.; 3 Department of Integrated Traditional Chinese and Western Medicine and NHC Key Laboratory of Transplant Engineering and Immunology, Frontiers Science Center for Disease-related Molecular Network, West China Hospital, Sichuan University, Chengdu, 610041, China.; 4 Center of Cellular and Molecular Diagnosis, Tulane University School of Medicine, 1430 Tulane Ave. New Orleans, LA 70112, USA.; 5 Department of Biochemistry & Molecular Biology, Tulane University School of Medicine, 1430 Tulane Ave. New Orleans, LA 70112, USA.

**Keywords:** severe acute pancreatitis, acute cardiac injury, lipid droplets, mitochondrial injury, metabolic disturbances

## Abstract

**Rationale:** Myocardial injury is a common and life-threatening complication of severe acute pancreatitis (SAP) and is driven primarily by metabolic disturbances. This study aimed to elucidate the pathogenesis of SAP-induced cardiac injury (SACI) and to identify effective therapeutic strategies.

**Methods:** Untargeted metabolomics and proteomics analyses were employed to identify metabolic pathways and proteins associated with myocardial injury in SACI mouse model. Histological and Western blot assays were used to assess lipid droplet (LD) accumulation, the expression of autophagy markers, and LD-autophagosome colocalization. The traditional Chinese medicine formula Taohong Siwu Decoction (THSWD) was tested for its therapeutic potential in a SACI mouse model and a SACI cardiomyocyte model established by incubating primary mouse cardiomyocytes with serum from the SACI mouse model. These SACI cardiomyocytes cultures were then treated with serum from control or THSWD-treated mice, with or without autophagy inhibitors, and analyzed for effects on lipophagy, mitochondrial structure and function, long-chain fatty acid metabolism, and oxidative stress.

**Results:** SAP-induced myocardial injury was characterized by disrupted lipid metabolism, leading to abnormal cardiomyocyte LD accumulation and structural and functional deficiencies in their mitochondria. THSWD treatment reduced LD accumulation, restored LD-autophagosome colocalization, and increased mitochondrial structural integrity, membrane potential, and fatty acid β-oxidation. However, these THSWD effects were abolished in the presence of an autophagy inhibitor, implying they occur via a lipophagy-dependent mechanism.

**Conclusion:** Excessive LD accumulation drives mitochondrial dysfunction, contributing to SAP-induced myocardial lipotoxicity. THSWD promotes lipophagy to mitigate lipid accumulation and restore mitochondrial function, and may serve as an effective therapeutic strategy for SAP-induced cardiac metabolic disorders and mitochondrial dysfunction.

## Introduction

Severe acute pancreatitis (SAP) is often accompanied by acute and severe myocardial injury and dysfunction, which contribute to its high mortality rate 1, 2. SAP-associated distal organ damage has traditionally been attributed to cytokine storms triggered by increased trypsin activation and elevated levels of proinflammatory factors, which ultimately lead to microcirculatory disorders, mitochondrial dysfunction, and apoptosis 3. However, the precise mechanisms underlying SAP-induced cardiac injury (SACI) remain unclear, and effective therapeutic interventions are urgently needed to improve patient outcomes.

The myocardium heavily relies on mitochondrial oxidative phosphorylation to meet its energy demands, and 50-70% of its ATP requirement is derived from fatty acid (FA) β-oxidation. Neutral lipids stored in lipid droplets (LDs) serve as a key reserve for myocardial energy metabolism 4. These LDs are dynamic organelles that regulate lipid metabolism via a mosaic of surface proteins, including perilipins (Plin1-5) 5. Circulating FAs taken up by the myocardium undergo β-oxidation to produce ATP or are stored in LDs as triglycerides (TGs), and subsequently hydrolyzed to provide substrates for mitochondrial metabolism 4, 6. An imbalance between myocardial FA uptake and oxidation can produce excessive LD accumulation and cytotoxic levels of ceramides, TGs, and other lipids, to disrupt cardiac function and contribute to metabolic stress 7, 8, although it is unclear whether this process is the primary driver of SAP-induced cardiac injury.

Selective autophagic degradation of LDs, or lipophagy, has emerged as a promising therapeutic approach for cardiometabolic disorders 9, as autophagosome interactions with selective autophagy receptors (SARs) on LDs can reduce their abundance and direct LD-derived cholesterol and FAs to mitochondria for ATP production 10. Lipophagy can reverse lipid accumulation in atherosclerosis and alleviate cardiac lipotoxicity in diabetic cardiomyopathy 9, 11, and is associated with reduced pancreatic lipid deposition and improved cellular organization and secretory function in animal models of pancreatic disease 12-14. However, studies have yet to explore the potential role of lipophagy in mitigating SAP-associated organ damage.

Taohong Siwu Decoction (THSWD), a traditional herbal medicine first recorded in the Golden Mirror of Medicine (Qing Dynasty), is derived from six herbs (*Rehmanniae Radix, Angelica sinensis Radix, Paeoniae Radix Alba, Chuanxiong Rhizoma, Carthami Flos, and Semen*), and is still widely used to increase blood circulation 15. THSWD has demonstrated therapeutic benefits in treating blood deficiency and blood stasis syndromes 16. Recent studies also suggest that THSWD can ameliorate myocardial injury via anti-inflammatory, antioxidative, anticoagulant, antifibrotic, and lipid-lowering effects, and can improve mitochondrial function by reducing excessive mitochondrial fission in coronary artery disease, myocardial infarction, ischemia-reperfusion injury, and atherosclerosis 17, 18. However, its potential therapeutic effects on SACI and its underlying mechanisms are unknown.

This study therefore characterized the effect of THSWD treatment on pathologic phenotypes detected in mouse and cell culture models of SAP-induced cardiac injury, and evaluated potential mechanisms responsible for these therapeutic effects. Our studies identified that Plin2, which plays an important role in lipid storage and metabolism, was differentially regulated in cardiac tissue of a SACI mouse model in the presence and absence of THSWD treatment, and formed potential interactions with several THSWD compounds and two mitochondrial proteins. Further, both Plin2 knockdown and THSWD treatment had similar effects to promote autophagy and the association of autophagosomes with lipid droplets, attenuate lipotoxicity and mitochondrial dysfunction. These findings provide strong experimental and mechanistic foundations for the clinical use of THSWD in the treatment of SAP-associated cardiac injury.

## Materials and Methods

### Reagents and antibodies

Taurocholic acid sodium salt (T4009), lipopolysaccharide (LPS) (L2880) and melatonin (M5250) were obtained from Sigma (USA). Chloroquine (CQ) (HY-17589A), bafilomycin A1 (Baf) (HY-100558) and atglistatin (ATGLi) (HY-15859) were obtained from MedChemExpress (USA). Anti-Plin2 (15294-1-AP), anti-Plin1 (27716-1-AP), anti-Plin3 (66523-1-Ig) and anti-carnitine palmitoyl-transferase1 (CPT1) antibodies (66039-1-Ig) were procured from Proteintech (Wuhan, China). The anti-LC3B antibody (18725-1-AP) was purchased from Thermo Fisher Scientific (USA). The anti-P62 antibody (A19700) was obtained from ABclonal Technology (Wuhan, China). The anti-COXI antibody (AF7002) was purchased from Affinity Biosciences (Jiangsu, China). The anti-TOMM20 antibody (D8T4N) was purchased from CST (USA). The anti-peroxisome proliferator-activated receptor-α (PPAR-α) antibody (sc-398394) was obtained from Santa Cruz Biotechnology (CA, USA). Antibodies against β-Actin (ab8226) and GAPDH (ab8245) were purchased from Abcam (Cambridge, UK). The mouse lipase (LPS) assay kit (A054) and free fatty acid (FFA) assay kit (A042-2-1) were obtained from Nanjing Jiancheng (Nanjing, China). Mouse interleukin-6 (IL-6) (88-7064) and tumor necrosis factor-α (TNF-α) (88-7324) enzyme-linked immunosorbent assay (ELISA) kits were purchased from Thermo Fisher Scientific (USA). The mouse brain natriuretic peptide (BNP) assay kit (RX202543M) and cardiac troponin I (cTnI) assay kit (RX202543M) were purchased from RuxinBio (Guangzhou, China). The MDA assay kit (S0131M) was purchased from Beyotime (Shanghai, China). The mouse catecholamine assay kit (ml001925-1) and ceramides assay kit (ml037499-1) were purchased from MlBio (Shanghai, China). The mouse 8-isoprostane assay kit (CB11115-Mu) was purchased from COIBO (Shanghai, China).

### Preparation of freeze-dried THSWD powder

THSWD was generated by crushing and mixing *Rehmanniae Radix, Angelicae Sinensis Radix, Paeoniae Radix Alba, Chuanxiong Rhizoma, and Persicae Semen* samples (9 g/each) with a* Carthami Flos* sample (6 g)*,* all of which were purchased from the Affiliated Hospital of Chengdu University of Traditional Chinese Medicine. These ingredients were processed in five times their volume of 50% ethanol for reflux osmotic extraction, and this extract was subsequently concentrated using a rotary evaporator and lyophilized to produce a 40% extract yield.

### Animals and animal study designs

Male C57BL/6 mice (20 ± 2 g) were acquired from Chengdu Dashuo Biological Technology Co., Ltd. (Chengdu, China). Mice were allowed to acclimate to the testing environment for one week (humidity, 50 ± 5%; temperature, 20 ± 2 °C), and then randomly divided into six groups (one sham control and five SACI mouse models with different treatments) and fasted for 12 h before use in an experimental procedure. Five of these groups were retrograde perfused via their pancreaticobiliary duct with a 2% taurocholic acid sodium salt solution (0.1 ml/100 g) to establish the SACI mouse model, using a previously described approach 19, 20, and the sixth group was perfused in the same manner with an equal volume of phosphate buffered saline (PBS) to establish the sham control group. With reference to *The Methodology of Pharmacological Experimen*t edited by Prof. Shuyun Liu et al (i.e., according to the mouse dose of approximately 9.1 times the clinical dose), the three SACI mouse groups were administered low, medium, or high THSWD doses (THSWD-L, 3.32 g/kg; THSWD-M, 6.63 g/kg; and THSWD-H, 13.26 g/kg), by gavage at 6 and 12 h after taurocholic acid perfusion (SACI+THSWD mice), and the fourth SACI mouse group were intraperitoneally injected with melatonin 12 h before taurocholic acid perfusion (20 mg/kg 21), and the SACI and the sham control mice did not receive treatment. All mice were sacrificed 24 h after treatment completion to collect arterial blood and pancreatic, cardiac lung, liver and intestine tissue samples for analysis. All animal study protocols were approved by the Experimental Animal Ethics Committee of Sichuan University of West China Hospital (Approval Number: 20240813003).

### Echocardiographic evaluation

Cardiac structure and function were evaluated in anesthetized mice by gently placing the probe of a VINNO6 LAB Ultrasound Imaging System (VINNO, China) on the left edge of the sternum between the 3rd and 4th ribs. This system was used to measure the left ventricular end-diastolic internal diameter (LVEDD), end-systolic internal diameter (LVESD), interventricular septal end-diastolic thickness (IVSd), and left ventricular posterior wall end-diastolic thickness (LVPWd). The ejection fraction (EF) and fractional shortening (FS) were then calculated to assess cardiac systolic function.

### Cardiac protein co-immunoprecipitation studies

Mouse cardiac tissue specimens were homogenized in RIPA buffer to generate protein extracts for immunoprecipitation studies. Protein A/G SureBeads (#161-4013/161-4023, Bio-Rad) (20 μL) were resuspended in 1 ml of PBST with 5 μL of nonspecific, TOMM20-specific, or COX1-specific IgG; shaken at room temperature for 1 h; washed three times with 1 mL of PBST; and mixed with aliquots of cardiac protein extracts for 2 h at room temperature. These beads were then recovered, washed three times with PBST, mixed with 40 μL of 1× protein loading buffer, and heated at 95 °C for 10 min to denature and release the captured proteins, which were subsequently detected by Western blot analysis.

### Physiological and biochemical index analysis

Amylase content and myocardial injury indices (e.g., TG, creatine kinase MB (CK-MB), and lactate dehydrogenase (LDH) levels were examined in mouse blood, pancreas, and cardiac tissue specimens and cell culture supernatants by an automatic biochemical analyzer (Chemray240, China). Lipase, TNF-α, IL-6, cTnI, BNP, FFA, MDA, catecholamine, ceramides and 8-isoprostane levels were measured using ELISA.

### Light and election microscope analyses

Cells and frozen mouse tissue sections employed for light microscopy analyses were fixed in 4% paraformaldehyde, embedded in paraffin blocks, and then sectioned to generate 4 µm-thick sections. Sections analyzed to measure mouse tissue histopathology scores were stained with hematoxylin for 5 min, and eosin for 1 min at room temperature, then dried and sealed and imaged on a light microscope for subsequent scoring using a published method 19. Sections analyzed for subcellular localization studies were incubated with fluorescent LC3B antibody (1:200, Thermo Fisher) to detect autophagosomes, BODIPY 493/503 (5 µM, Thermo Fisher) to detect lipid droplets (LDs), and DAPI (C0065-10ML, Solarbio) to detect cell nuclei. Images were captured using an Olympus fluorescence microscope (Olympus, Japan), and the colocalization of the LC3B and LD signal distributions quantified by Image J software analysis was evaluated by their Pearson's correlation coefficient (PCC).

Primary cardiomyocytes analyzed by a long-chain FA (LCFA) tracking assay were cultured with 1 µM Bodipy 558/568 C12 (Red C12, D3835, Thermo Fisher) for 16 h to label their LCFAs, as previously described 22. Cell cultures were then incubated with 100 nM PK Mito Deep Red (PKMDR-2, Genvivotech) for 15 min to label their mitochondria 23, and then incubated with 5 µM Bodipy 493/503 (D3922, Thermo Fisher) for 15 min to label their LDs. Stained cell images were captured with a Zeiss LSM 980 confocal microscope (Carl Zeiss), and the colocalization of LCFAs with the LDs and mitochondria of these cells was assessed using Manders' colocalization coefficients (MCCs) and ImageJ software.

Cardiac tissue specimens analyzed by transmission electron microscopy to evaluate mitochondrial structure changes were prefixed using a 2.5% glutaraldehyde solution, dehydrated with a graded acetone series, embedded, and cut into 60-90 nm ultrathin sections, which were placed on a copper mesh, and stained with uranium acetate for 10-15 min, and lead citrate for 1-2 min. Images were collected using a JEM-1400FLASH (Japan) transmission electron microscope.

### THSWD compound identification and pharmacologic network analysis studies

THSWD constituent compounds and THSWD-derived compounds in the serum of THSWD-treated mice were identified by ultra-performance liquid chromatography-mass spectrometry (UPLC-MS/MS). Mouse serum samples prepared for this analysis were generated by dissolving THSWD in 5-fold distilled water, after which healthy mice were gavaged twice, at six hours intervals, with THSWD (6.63 g/kg) and sacrificed 12 h after the final gavage to collect blood for the serum analysis study. THSWD and serum specimens collected from THSWD-treated mice were mixed with 60% methanol spiked with an internal standard peptide, centrifuged, and subsequently analyzed by UPLC-MS/MS. Mobile phase A: 0.1% formic acid-water. Mobile phase B: 0.1% formic acid-acetonitrile. The PubChem database and Swiss Target Prediction database (http://www.swisstargetprediction.ch/) were used to identify chemical associated with detected UPLC-MS/MS peaks, and these were assigned Uniprot database (https://www.uniprot.org/) identifiers. These compound identifiers were was then analyzed with Cytoscape software to identify potential interactions. Kyoto Encyclopedia of Genes and Genomes (KEGG) and Gene ontology (GO) analyses were also performed using the DAVID database (http://david.ncifcrf.gov/) (p <0.05) to identify potential biochemical pathway interactions. THSWD compounds that were identified in this analysis were imported into the PubChem database (https://pubChem.ncbi.nlm.nih.gov/) to obtain their chemical structures.

### Pharmacokinetics of THSWD components in plasma and cardiac tissue

Plasma and cardiac tissue homogenates collected and processed at 10 and 30 min and 1, 3, 6, 12, and 24 h after THSWD administration were rapidly thawed at room temperature and vortexed ensure their homogeneity. Aliquots of these mouse samples (30 µL) were added to microfuge tubes and mixed with 120 µL of acetonitrile, vortexed for 10 s, centrifuged at 13000 rpm for 10 min, and the supernatants were analyzed using by UPLC-MS/MS using an ACQUITY UPLC HSS C18 1.7 um, 2.1 ×100 mm chromatographic column. The compounds (standards: Amygdalin: HY-N0190, Angelicin: HY-N0763, Paeoniflorin: HY-N0290, Picrocrocin: HY-N4114, Rehmannioside: HY-N0912, and Senkyunolide F: HY-N0774 were obtained from MedChemExpress) were determined by using the multistage reaction mode (MRM) detection mode.

### Preparation of THSWD-containing and SACI mouse serum for cell culture experiments

Healthy male C57BL/6 mice were administered a medium THSWD dose (6.63 g/kg) by gavage twice a day at 6 to 8 h intervals (morning and afternoon) for seven consecutive days or retrograde perfused via their pancreaticobiliary duct with a 2% taurocholic acid sodium salt solution (0.1 ml/100 g) and sacrificed at 30 min or 1 h after the last the last gavage to collect THSWD containing serum (THSWD serum) or SACI mouse serum, respectively. Arterial blood was collected from these mice immediately after sacrifice and processed for serum, which was then filter sterilized using a 0.22 μm microporous filter and stored at -80 °C until use.

### Mouse primary cardiomyocyte culture studies

Neonatal (1-3 days old) C57BL/6 mice sacrificed by over anesthesia were immersed twice for 15 min in 75% ethanol, after which their thoracic cavities were opened to collect their hearts, which were washed twice with PBS. These hearts were then cut into small pieces (~2 mm^3^), added to a 0.25% trypsin solution (25200056, Gibco) (0.5 ml/3 hearts), and incubated at 4 °C for 6 h, after which the digestion was terminated by the addition of DMEM (6124210, Gibco) containing 10% FBS (1414426, Gibco) and 1% penicillin/streptomycin (15070063, Gibco). These samples were then digested for 8 min at 4 °C after the addition of type II collagenase (17101015, Gibco) (0.5 ml/3 hearts), then filtered through a 100 µm filter, after which their filtrates were evenly spread on 100 mm^2^ Petri dishes that were transferred to a 37 °C / 5% CO2 incubator for differential apposition (30 min each time), and then the supernatant (containing a large number of cardiomyocytes) was collected for different interventions.

These primary mouse cardiomyocyte cultures were supplemented with 1 μg/mL LPS plus 5%, 10%, 15%, or 20% SACI mouse serum and cultured for 6, 12, or 24 h to determine the minimum serum and time conditions required to significantly reduce cell viability, as evaluated by their CCK-8 assay results (24033227, Biosharp, China). After establishing the SACI model, the supernatant was discarded and 0, 2, 5, 10, 15, 20, or 25% THSWD serum was added, as well as untreated sham-control cardiomyocyte cultures incubated for 24 h to evaluate the therapeutic and cytotoxic effects of THSWD serum. After the SACI model was established, supernatants were discarded and SACI cardiomyocytes were incubated with 20% THSWD for 2, 4, 6, 8, 10, 12, or 18 h to determine optimal time for THSWD intervention. According to the previous method, 250μM palmitate (PA) 24 was used to establish a myocardial lipotoxicity model. 100 μmol/L paeoniflorin (HY-N0290, MedChemExpress) 25 and 1 mmol/L senkyunolide F (HY-N0774, MedChemExpress) 26, and the combination of these two monomers was co-incubated with the SACI model and the PA model.

### Plin2 siRNA transfection studies

For Plin2 siRNA transfection studies, cardiomyocyte cultures were transfected for 48 h with Plin2-specific siRNAs (Table S1) synthesized by Starvio Biotechnology Company Limited (Shanghai, China) using Starvio^PM^ siRNA Primary Cell Transfection Reagent (T11008) according to the supplied protocol.

### Calcium content measurement

Cardiomyocyte intracellular calcium ion [Ca^2+^]_i_ concentrations were measured using a Fluo-4 AM Assay Kit (Beyotime, S1060S), and changes in fluorescence intensity (485 nm excitation, 528 nm emission) were recorded using a BioTeK Synergy Mx fluorescence microplate reader (USA).

### Mitochondrial membrane potential assay

Mitochondria were isolated from fresh cardiac tissue specimens using a mitochondria extraction kit (SM0020-50T; Solarbio). Mitochondrial membrane potential was measured in cardiac tissue mitochondria samples or fresh cardiomyocytes by a JC-1 assay kit (M8650, Solarbio), and the signal from these specimens was quantified using a BioTeK SynergyMx fluorescence microplate reader (USA) or an Olympus IX83 fluorescence microscope (Japan), respectively.

### Measurement of reactive oxygen species (ROS) levels and ATP production

Intracellular and mitochondrial ROS levels and cardiomyocyte or cardiac tissue ATP production were analyzed using Reactive Oxygen Species Assay Kits (S0033S, Beyotime), Mitochondrial Superoxide Fluorometric Assay Kits (E-BC-F008, Elabscience), and ATP Content Assay Kits (BC0300, Solarbio), using a BioTeK SynergyMx fluorescence microplate reader (USA) to quantify assay signal and an Olympus IX83 fluorescence microscope (Japan) to capture representative fluorescence images.

### Western blotting

Cardiac tissue and cultured cardiomyocyte samples were homogenized in RIPA buffer, and the total protein concentrations of these protein extracts were quantified with a bicinchoninic acid kit (P0012S, Beyotime). Supernatant proteins of 10-20 µg/lane were size-fractioned on 10% SDS‒PAGE gels, transferred to polyvinylidene difluoride membranes (Millipore, USA), which were blocked (EpiZyme Biotech, Shanghai, China) by shaking on a shaker for 10 min at room temperature, and then and incubated overnight at 4 °C with antibodies specific for LC3B (1:1000), P62 (1:1000), Plin2 (1:2000), Plin1 (1:2000), Plin3 (1:10000), TOMM20 (1:1000), COXI (1:2000), CPT1 (1:2000), PPAR-α (1:1000), GAPDH (1:10000) or β-actin (1:10000). Finally, an innovative imaging system from Bio-Rad (Hercules, CA, USA) was used to visualize the blots.

### Protein and molecular docking simulations

The European Bioinformatics Institute (EBI), Swiss Institute of Bioinformatics (SIB), and Protein Information Resource (PIR) databases were searched to identify Plin2, TOMM20, and COXI protein structures. Protein‒protein docking simulations were performed using the HDOCK server to display the protein and protein binding processes as surface models. The MM/GBSA server was used to calculate free binding energies, and the PLIP interaction analysis platform was used to systematically characterize and analyze the binding interfaces of protein‒protein complexes. Structures of Amygdalin (CAS:29883-15-6), Angelicin (CAS:523-50-2), Paeoniflorin (CAS:23180-57-6), and Picrocrocin (CAS:138-55-6) were obtained from the PubChem database, and the 3D structures of Rehmannioside D (CAS:81720-08-3) and Senkyunolide F (CAS:94530-84-4) were obtained in SDF format. Molecular docking analysis was performed to assess the affinity of candidate drug compounds for their targets. AutodockVina 1.2.2 was used to determine the predicted binding positions of these candidate drugs on their target proteins, and the binding energies of the optimal protein‒ligand complex interactions. Binding interfaces of the prediced protein‒ligand complexes were systematically analyzed using PLIP and LigPlus, and interaction-related details were evaluated with PyMOL 2.5 software.

### Proteomic analysis

Mouse cardiac tissue samples (50 mg each) were used to generate protein extracts that were processed to remove contaminant using Pierce™ C18 Spin Tips (Thermo Fisher, USA). Chromatographic separations were performed using an Easy-nLC1200 system (Thermo Fisher, USA) and analyzed with an Orbitrap Fusion Lumos mass spectrometer (Thermo Fisher, USA), and the resulting data was analyzed against the UniProt database to identify and quantify detected proteins. Differentially expressed proteins (DEPs) were defined as those that had absolute log2 fold change (|log2 FC|) values ≥ 0.58 and adjusted p-values < 0.05. Detailed data analyses reported in this study were performed online at the APExBIO website (https://analize.cloud.apexbio.cn/).

### Untargeted metabolomics analysis

Serum (50 µL each) and cardiac tissue (50 mg each) samples from the sham, SACI and SACI+THSWD mouse groups were obtained for metabolomic analysis (n = 6/group) and analyzed as described in a previous study 19. This data was processed by orthogonal partial least squares-discriminant analysis (OPLS-DA). Differentially abundant metabolites (DEMs) were subsequently detected based on their independent samples t-test values (p <0.05) and variable significance in projection (VIP) scores (≥ 1). Detailed data analyses reported in this study were performed online at the APExBIO website (https://analize.cloud.apexbio.cn/).

### Seahorse metabolic flux analysis

Primary cardiomyocyte specimens were incubated with Seahorse XF basal medium for 1 h and then sequentially incubated with oligomycin (1.0 μM), FCCP (1.0 μM), and rotenone/antimycin A (0.5 μM) to measure their oxygen consumption rates (OCR), basal mitochondrial respiration and maximal respiratory capacity using a Seahorse XFe24 extracellular flux analyzer and a Seahorse XF Cellular Stress Assay Kit (#103015-100, Seahorse Bioscience, USA). These data were normalized according to the BCA protein assay results determined for aliquots of these samples (pmol O_2_/min/μg protein).

### Statistical analysis

All data were analyzed using GraphPad Prism 9 software. Results are presented as mean ± standard error of the mean (SEM) values, and tested for normality. Independent samples t-tests were used for comparisons between two groups; one-way analysis of variance (ANOVA) or two-way ANOVA were used for three or multiple groups with Tukey's multiple comparisons tests. Kruskal-Wallis H test was used for non-normally distributed or inhomogeneous variance data. P-values <0.05 were considered significant. At least three samples were analyzed per experimental group.

## Results

### THSWD attenuates pathologic cardiac tissue changes linked with SACI metabolic dysregulation

**Figure 1:**
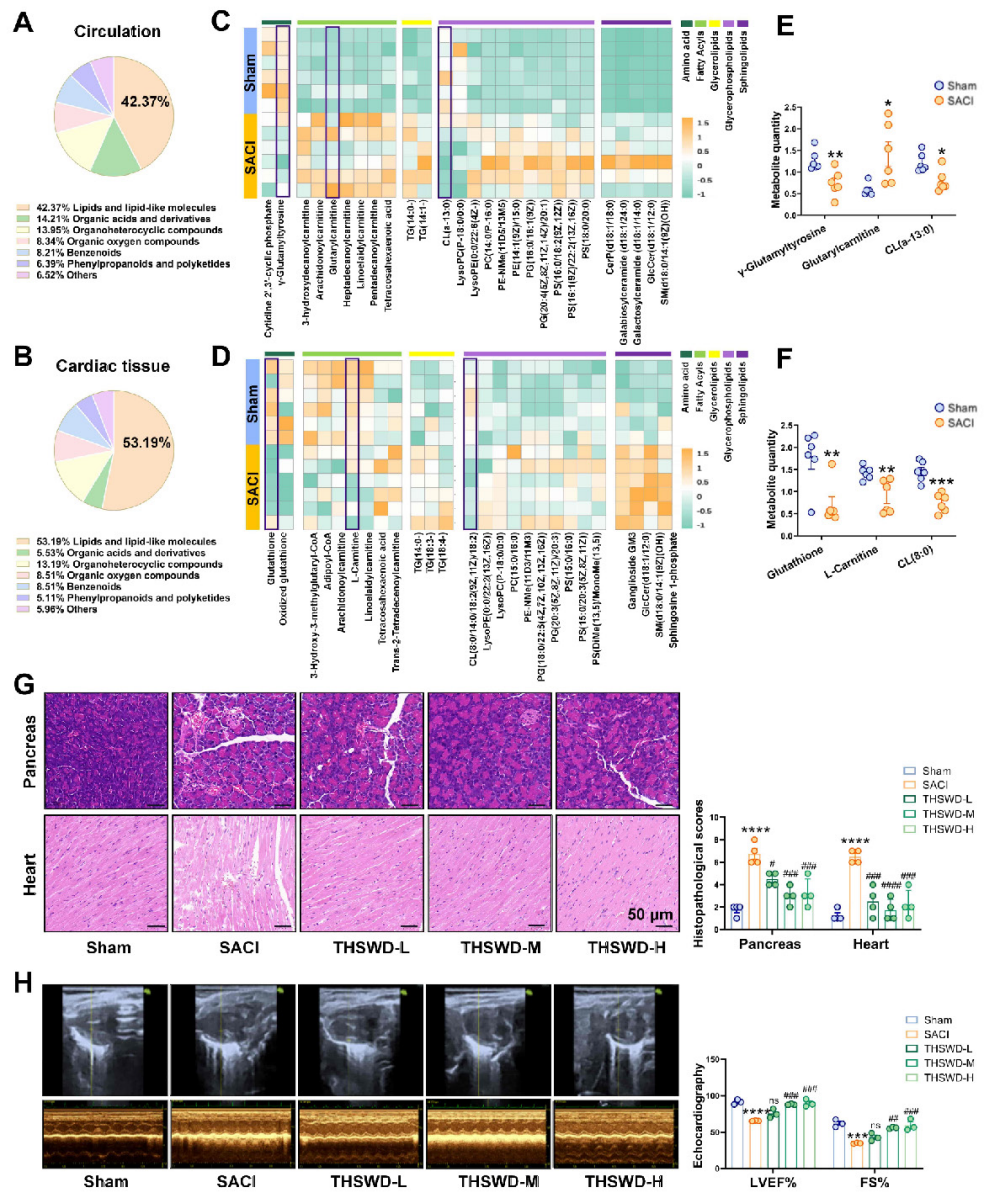
** THSWD attenuates pathologic cardiac tissue changes linked with SACI metabolic dysregulation.** (**A**, **B**) Pie charts of the percentage of different types of metabolites detected in circulation and cardiac tissue samples from sham and SACI model mice. (**C**, **D**) heatmaps and (**E**, **F**) statistical charts of specific differences in lipid metabolite abundance detected in circulation and cardiac tissue samples from sham and SACI model mice (n = 6/group). (**G**) Representative images and histopathological scores of H&E-stained pancreas and cardiac tissue samples of sham control and SACI mice treated with or without low, medium or high THSWD doses (THSWD-L, THSWD-M, or THSWD-H) (scale bar = 50 μm; n = 4/group). (**H**) Mouse echocardiogram images and LVEF and FS values were calculated for each group (n = 3/group). Results are presented as mean ± SEM values. *p < 0.05, **p < 0.01, ***p < 0.005, ****p < 0.0001 vs. sham; #p < 0.05, ##p < 0.01, ###p < 0.005, ####p < 0.0001 vs. SACI. ns: nonsignificant vs. SACI.

Mice were perfused with taurocholic acid sodium salt solution to induce SACI 23 or with PBS to generate a sham control. Untargeted metabolomics analysis of serum and cardiac tissue samples from these SACI model versus sham control mice revealed altered abundance in the circulation and cardiac tissues (42.37% and 53.19%) were associated with lipid metabolism processes, and substantial SACI-associated increases in FA and lipid content paired with decreased abundance of amino acids, glutathione and acetyl-coenzyme A metabolites, resulting in strong discrimination of these samples by differences in their lipid and lipid-like molecule profiles (Figures 1A-D, S1A-B). These included increased serum and decreased cardiac tissue levels of long-chain acylcarnitines in the SACI versus sham mouse groups, and reduced levels of a mitochondrial membrane-associated lipid, cardiolipin (CL) (Figures 1E-F), suggestive of disrupted cardiac FA β-oxidation and mitochondrial damage resulting in an imbalance between energy demand and production.

SACI mice were then treated with or without low, medium, or high THSWD doses, to evaluate the effects of THSWD on SACI-induced systemic tissue injuries. Most THSWD doses reduced the histopathology scores of SACI mouse pancreas, heart, lung, liver and intestine samples; their serum amylase, lipase, proinflammatory cytokine, cardiac injury marker levels, catecholamine secretion and FFA levels; and their left ventricular ejection fraction (LVEF) and fractional shortening (FS) values (Figures 1G-H, S1C-D). However, THSWD effects on lung histopathology and some cardiac injury markers were dose dependent, with the best results observed in the medium-dose group. No significant histopathology or proinflammatory effects were observed when control mice were treated with medium-dose THSWD, implying this dose was nontoxic (Figures S1E-F). Also compared to melatonin, an effective control drug for septic cardiomyopathy 21, 27, medium-dose THSWD could serve more effective SACI treatment in the SACI mouse model (Figure S1G).

### THSWD regulates cardiac expression of genes associated with lipid storage and metabolism

**Figure 2:**
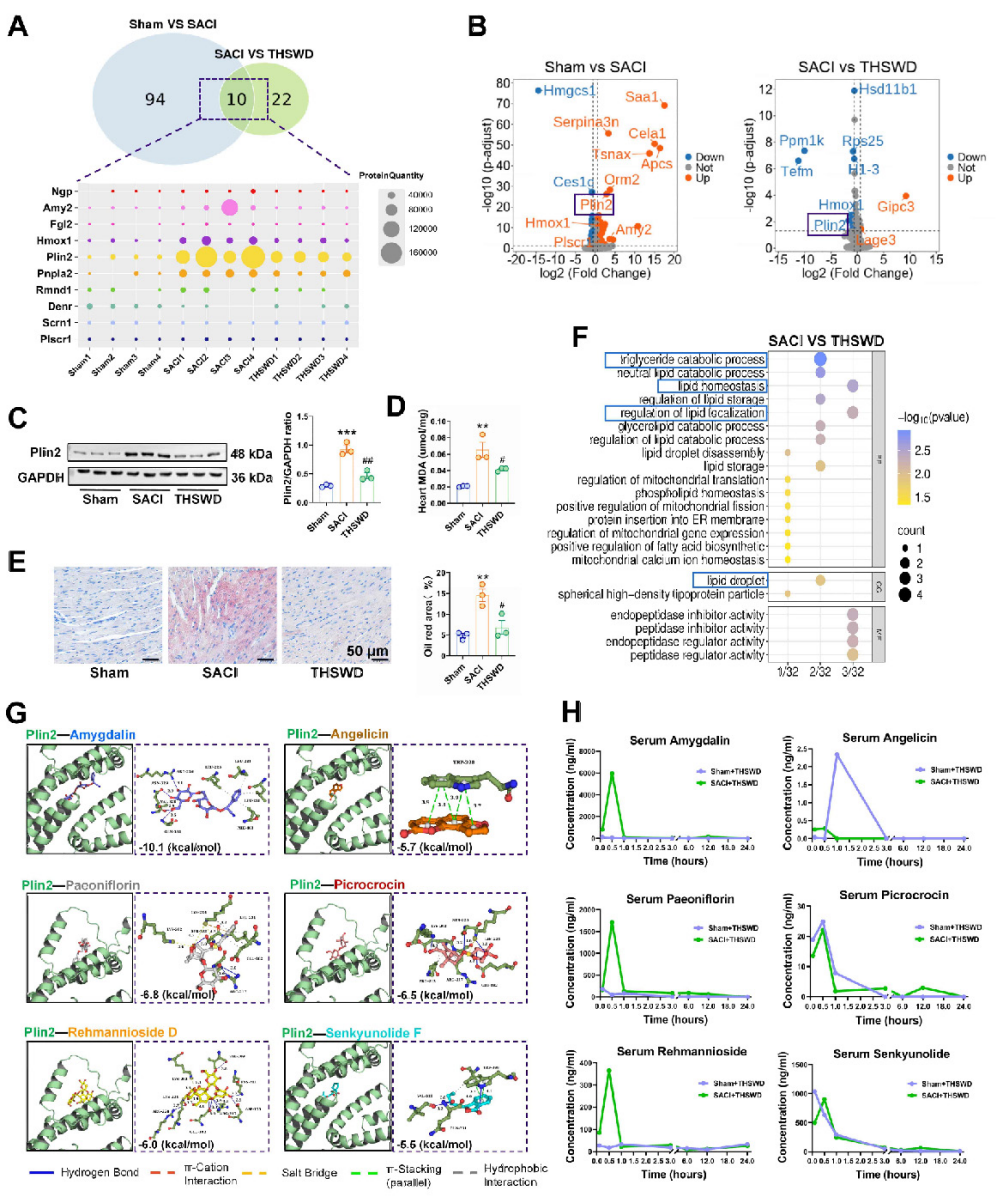
** THSWD regulates cardiac expression of genes associated with lipid storage and metabolism.** (**A**) Venn diagram of cardiac tissue DEPs in sham vs. SACI model mice and SACI vs. SACI+THSWD mice, and a heatmap of the relative expression of 10 DEPs shared among these groups (n = 4/group). (**B**) Volcano plots of DEPs of sham vs. SACI mice and SACI+THSWD vs. SACI mice (n = 4/group). (**C**) Western blot and quantitative analysis of Plin2 expression in cardiac tissue of sham, SACI, and SACI+THSWD mice (n = 3/group). (**D**) Cardiac tissue MDA levels and (**E**) representative images and quantitative analyses of Oil Red O staining in these groups (n = 3/group). (**F**) GO analysis of cardiac tissue DEPs of SACI+THSWD vs. SACI model mice (n = 4/group). (**G**) Molecular dynamics simulation of potential Plin2 interactions with six major THSWD components using HDOCK software. (**H**) Serum concentration-time profiles of the six major THSWD components in sham and SACI mice at 10 and 30 min, and 1, 3, 6, 12, and 24 h after THSWD administration. Results are presented as mean ± SEM values. **p < 0.01, ***p < 0.005 vs. sham; and #p < 0.05, ##p < 0.01 vs. SACI.

OPLS-DA of differentially expressed proteins (DEPs) in cardiac tissue of sham and THSWD-treated and untreated SACI mice revealed a strong classification effect (Figure S2A). Comparative analyses of the sham vs. SACI mouse data and the THSWD-treated vs. untreated SACI mouse data identified 104 DEPs and 32 DEPs, respectively, including 10 DEPs (Ngp, Amy2, Fgl2, Hmox1, Plin2, Pnpla2, Rmnd1, Denr, Scrn1, and Plscr1) detected in both comparisons (Figure 2A). However, only two of these 10 proteins revealed differential expression in volcano plots of this data (Figure 2B): Hmox1 and the LD protein Plin2, which are involved in heme degradation and lipid storage and metabolism, respectively. Notably, reduced Plin2 expression in the treated vs. untreated SACI mice aligned with reductions in cardiac lipid deposition and peroxidation phenotypes of these mice (Figures 2C-E).

Sham vs. SACI mouse cardiac tissue DEPs were associated with the GO terms “fatty acid binding, lipid storage, and lipid homeostasis”, whereas SACI vs. SACI+THSWD mouse DEPs were associated with “triglyceride catabolic process, lipid homeostasis, regulation of lipid localization and lipid droplet” GO terms (Figures 2F, S2B). These results suggest that THSWD's effects to attenuate increased cardiac Plin2 expression in SACI mice could directly enhance lipid catabolism and reduce LD accumulation, consistent with attenuated cardiac lipid deposition and peroxidation changes detected in SACI+THSWD mice that appear to restore cardiac lipid homeostasis (Figures 2C-E).

Subsequent UPLC‒MS/MS analysis of THSWD detected 18 major components, 16 of which were also detected in the sera of sham mice treated for one week with a medium dose of THSWD (Table S2, Figures S3A‒D). Simulation studies revealed that Plin2 could potentially interact with six of the major compounds identified in THSWD (Figure 2G), and that serum and cardiac tissue levels of these compounds peaked within 1 h after THSWD treatment (Figures 2H, S3E), suggesting that one or more of them could directly interact with Plin2 to alter its regulatory effects on lipid storage and metabolism in SACI mouse cardiac tissue. Network pharmacology analysis detected 170 potential therapeutic targets for these 16 THSWD components (Figure S3F). Subsequent gene ontology analysis revealed these targets were associated with the functional terms inflammatory response, plasma membrane, and ATP binding (Figure S3G), whereas KEGG analysis revealed associations with TNF signaling pathway, HIF-1 signaling pathway, and lipid and atherosclerosis terms (Figure S3H). These results thus also suggest that THSWD may exert its therapeutic effects on SACI through pathways that regulate lipid metabolism.

### THSWD promotes lipophagy and attenuates SACI-induced mitochondrial damage

**Figure 3:**
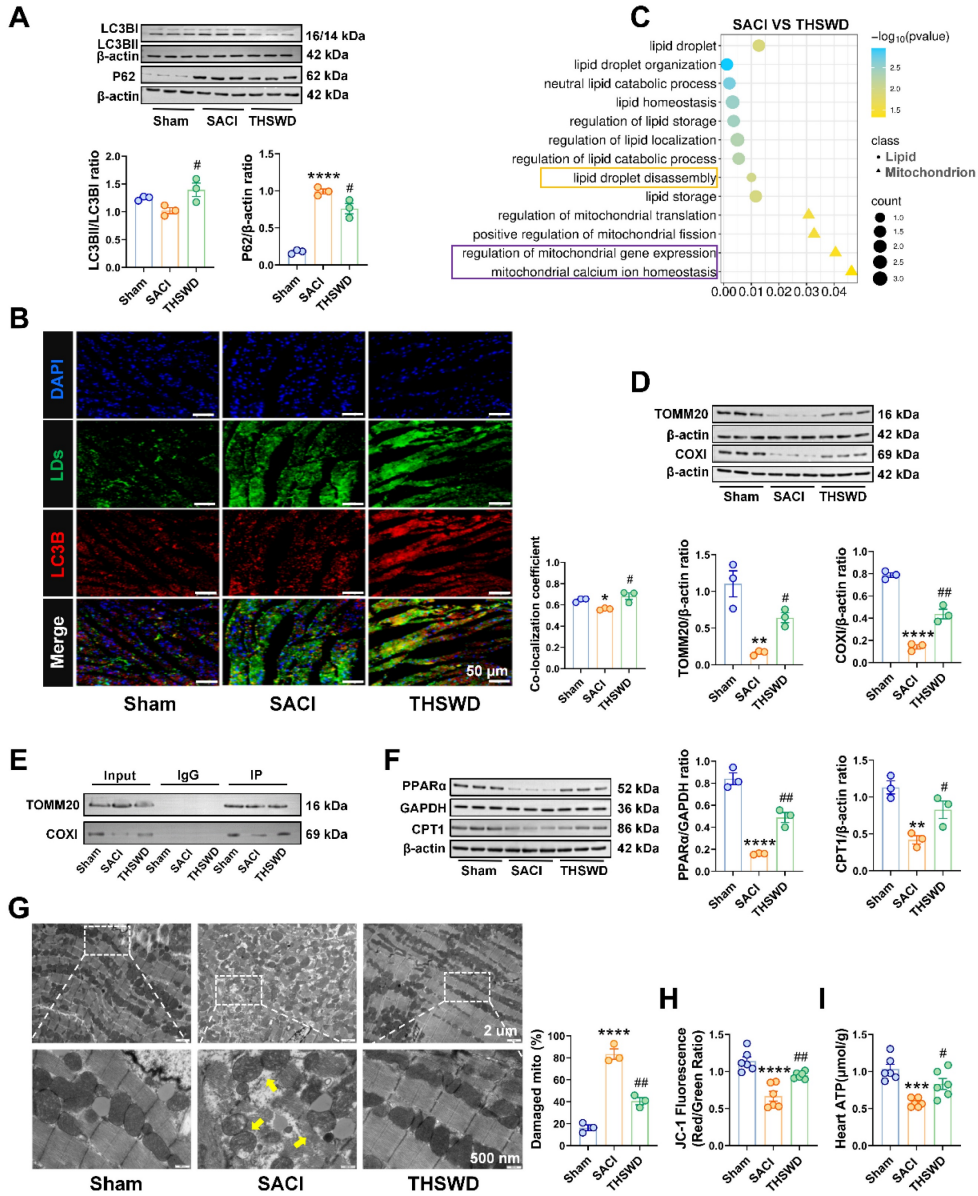
** THSWD promotes lipophagy and attenuates SACI-induced mitochondrial damage.** (**A**) Western blot and quantitative analysis of LC3B and P62 protein expression in cardiac tissue of the sham, SACI, and SACI+THSWD groups (n = 3/group). (**B**) Representative images and colocalization of LC3B (red), LDs (green), and nuclear (blue) fluorescent signals in the cardiac tissue of these mouse groups (scale bar = 50 μm; n = 3/group). (**C**) GO analysis of DEPs in the cardiac tissues of SACI+THSWD vs. SACI model mice (n = 4/group). (**D**) Representative Western blots and quantification of TOMM20 and COXI expression in these groups (n = 3/group). (**E**) Co-IP analysis of Plin2 interactions with TOMM20 and COXI. (**F**) Representative Western blot image and quantitative analysis of PPAR-α and CPT1 expression in cardiac tissue of the sham, SACI, and SACI+THSWD groups (n = 3/group). (**G**) Representative transmission electron microscopy images of cardiac tissue mitochondria and their structural defects in these groups (scale bar = 2 μm; 500 nm; n = 3/group; yellow arrows: damaged mitochondria). (**H**) Membrane potential (JC-1 fluorescence: red/green ratio) of isolated cardiac mitochondria and (I) cardiac tissue ATP levels in each mouse group (n = 6/group). Results are presented as mean ± SEM values. **p < 0.01, ***p < 0.001, ****p < 0.0001 vs. sham; #p < 0.05, ##p < 0.01 vs. SACI.

LDs are highly dynamic organelles that can be regulated by lipophagy, a specific form of autophagy, that may influence LD homeostasis. Western blot analysis of the cardiac expression of the autophagy-associated proteins LC3B and P62, detected a marked P62 increase in SACI vs. sham control mice, consistent with attenuated autophagy, and a LC3BII/LC3BI increase and a P62 decrease in SACI+THSWD vs. SACI mice, consistent with a restoration of autophagy (Figure 3A). We also detected a marked reduction in LC3B protein colocalization with LDs in SACI mouse cardiac tissue, which was reversed in SACI+THSWD, suggesting that SACI and THSWD treatment had opposing effects to suppress and restore cardiac lipophagy (Figure 3B), consistent with the enrichment of the “lipid droplet disassembly” GO term upon proteomic analysis of SACI vs. SACI+THSWD mouse cardiac tissue (Figure 3C).

Excessive LD accumulation can cause lipotoxicity, which often manifests as mitochondrial dysfunction. Proteomic analysis of SACI vs. SACI+THSWD mouse cardiac tissue also detected an enrichment of the GO terms “regulation of mitochondrial gene expression” and “mitochondrial calcium ion homeostasis” (Figure 3C), suggesting that THSWD could also have a therapeutic effect on mitochondrial function in SACI mouse cardiac tissue.

To evaluate potential THSWD effects on mitochondria function, we analyzed the cardiac levels of the mitochondrial membrane transporter TOMM20 and the mitochondrial respiratory complex protein COXI to evaluate potential changes in mitochondrial biogenesis or function 28, 29. This analysis detected marked decreases in cardiac TOMM20 and COXI protein levels in SACI vs. sham mice, and that these decreases were strongly attenuated in SACI+THSWD mice (Figures 3D). Both these proteins were subsequently found to coprecipitate with Plin2, suggesting the potential for direct LD-to-mitochondrion interactions that could influence cardiac lipotoxicity in SACI mice (Figures 3E). Consistent with an effect to inhibit SACI-induced cardiac lipotoxicity, THSWD treatment was observed to reverse decreased expression of two proteins involved in FA metabolism, PPAR-α and CPT1; reduce mitochondrial structural damage (swelling, fractures, and partial dissolution of mitochondrial cristae); and improve mitochondrial membrane potential and ATP production decreases detected in SACI mouse cardiac tissue (Figures 3F-I). Taken together, these results suggest that a THSWD effect to increase lipophagy in SACI mouse cardiac tissue could have attenuated LD abundance and LD-mitochondria interactions to reduce SACI-induced cardiac lipotoxicity.

### Plin2 knockdown mimics THSWD effects to attenuate SACI-associated cardiomyocyte lipotoxicity

Primary mouse cardiomyocytes were next cultured with LPS and increasing concentrations of SACI mouse serum to establish a cell culture model of SACI cardiotoxicity, and 6 h incubation with 10% SACI mouse serum was determined to be sufficient to consistently induce cytotoxicity in this SACI cardiomyocyte model (Figures S4A-B). Subsequent analysis of standard and SACI cardiomyocyte cultures incubated with or without serum obtained from THSWD-treated sham control mice revealed that THSWD-serum had no effect on cell viability in normal cardiomyocyte cultures, but dose-dependently attenuated SACI cardiomyocyte death, with the maximum effect observed after 10 h of treatment with 20% THSWD-serum (Figures S4C-E), which was used in all subsequent studies.

**Figure 4:**
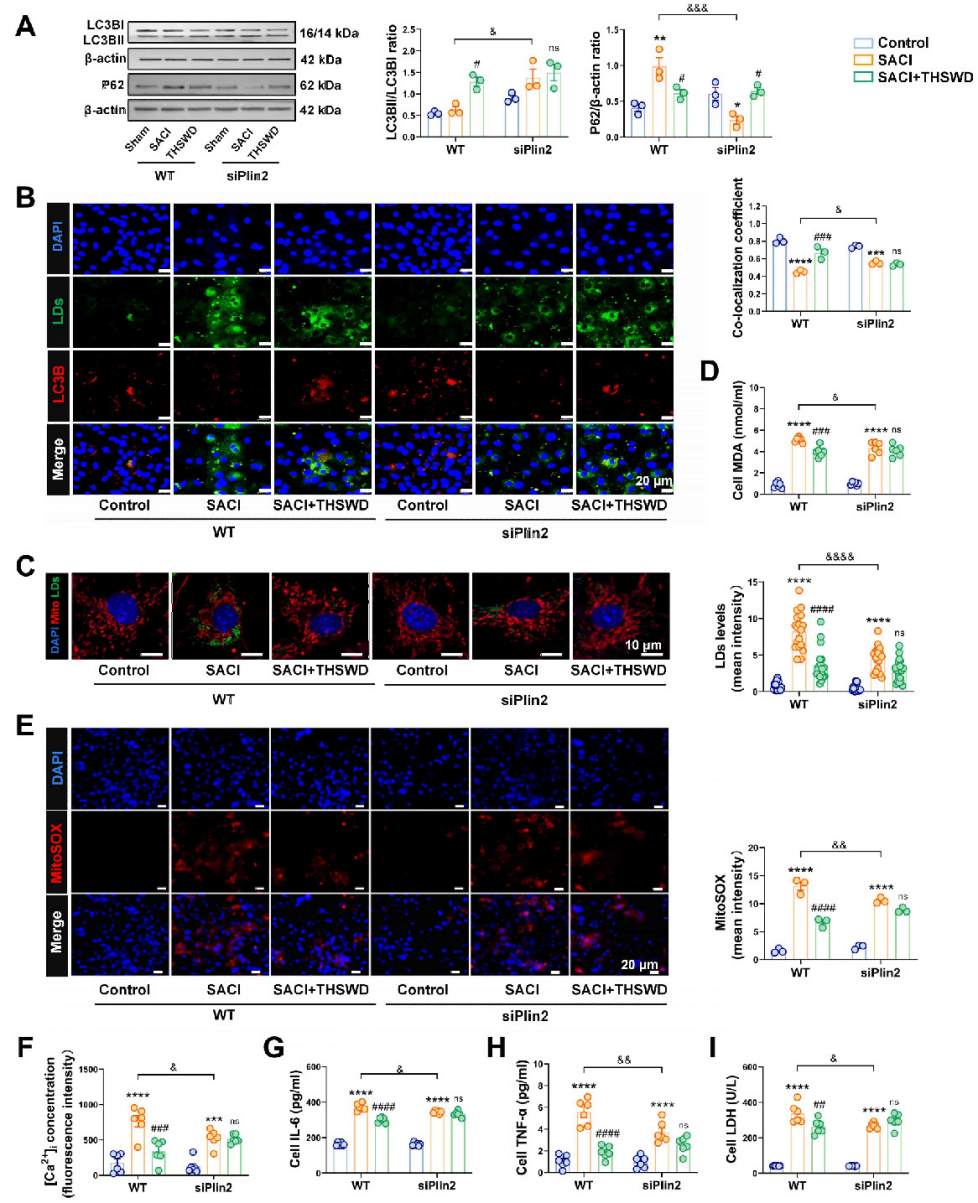
** Plin2 knockdown mimics THSWD effects to attenuate SACI-associated cardiomyocyte lipotoxicity.** (**A**) Western blot and quantification of LC3B and P62 protein expression in the indicated cardiomyocyte cultures (n = 3/group). (**B**) Colocalization of LC3B (red), LD (green), and nuclear (blue) fluorescent signals in these groups (scale bar = 20 μm; n = 3/group). (**C**) Representative images of mitochondrial (red), LD (green) and nuclear (blue) fluorescent signals in these groups and quantification of their LD signal (scale bar = 10 μm; n = 18 cells/group). (**D**) Supernatant MDA levels in these cultures (n = 6/group). (**E**) Representative images of MitoSOX signal (red) and its quantification in these groups (scale bar = 20 μm; n = 3/group). (**F**) Intracellular calcium ion ([Ca2+]i) levels in these cardiomyocyte cultures (n = 6/group). (G-I) IL-6, TNF-α and LDH levels measured in the supernatants of these cultures (n = 6/group). Results are presented as mean ± SEM values. **p < 0.01, ****p < 0.0001 vs. Control; #p < 0.05, ##p < 0.01, ###p < 0.005, ####p < 0.0001 vs. SACI; &p < 0.05, &&p < 0.01, &&&p < 0.005, &&&&p < 0.0001 vs. wild type (WT) SACI; ns: nonsignificant vs. siPlin2 SACI.

Since THSWD may exert its observed cardioprotective by regulating Plin2 activity, based on our previous data, we next analyzed the effect of a siRNA-mediated Plin2 knockdown on SACI associated cardiac phenotypes. Plin2 knockdown increased LC3B expression and reduced P62 expression in SACI cardiomyocytes, with a slight increase in LC3B and LD co-localization, consistent with an overall effect to increased lipophagy, despite a minor decrease of lipid droplets abundance (Figure 4A-C). At the same time, Plin3 was compensatorily upregulated, consistent with the fact that lipid droplets were still present; although changes in Plin1 were not significant 30 (Figure S5A-B). In addition, the change in Plin3 expression between the THSWD and SACI groups was not significant after knockdown of Plin2, suggesting that THSWD more often targets Plin2 (Figure S5C). Moreover, Plin2 knockdown reduced the accumulation of the toxic lipid ceramides in SACI cardiomyocyte, and decreased the lipid peroxidation markers: 8-isoprostane and MDA (Figures 4D, S5D-E). Plin2 knockdown also reduced SACI cardiomyocyte mitochondrial and intracellular ROS production, [Ca^2+^]_i_ levels, and proinflammatory cytokine secretion (Figures 4E-I, S5F), while increasing their mitochondrial membrane potentials and ATP production (Figures S5G-H). Notably, Plin2 knockdown tended to reduce or abolish differences between the SACI vs. SACI+THSWD cardiomyocyte phenotypes, implying that both the siRNA and THSWD effects were mediated by attenuating SACI cardiomyocyte Plin2 expression.

In addition, we screened two major monomeric components of THSWD, paeoniflorin and senkyunolide F, and used these two monomers alone or in combination to intervene in cardiomyocyte models continuously exposed to SACI serum and in a lipotoxic cardiomyocyte model constructed with palmitate (PA) to validate the direct effects of THSWD on cardiomyocytes. The results showed that the monomer components of THSWD increased cell viability and still decreased the level of inflammatory factor TNF - α and the expression of key LD protein Plin2 after 12 h of co-incubation with the SACI model and 6 h of co-incubation with the PA model (Figure S6A-F). Notably, the monomer combination intervention was superior to the single - monomer intervention (Figure S6C-F). The above results suggest that THSWD can act directly on cardiomyocytes to exert its efficacy in the presence or absence of potential pancreatic factor influences, as evidenced directly by its monomer efficacy.

### THSWD attenuates lipotoxicity induced by SACI cardiomyocyte LDs by promoting lipophagy

SACI cardiomyocytes were next treated with the autophagy inhibitors chloroquine (CQ) or bafilomycin A1 (Baf) 27, 28 or the lipolysis inhibitor atglistatin (ATGLi) 29 to evaluate whether THSWD increased lipophagy or lipolysis to reduce LD accumulation (Figure S7A). CQ and Baf inhibited autophagy and decreased the LD-autophagosome colocalization in the presence or absence of THSWD, although the LD-autophagosome dissociation effect was greater in the absence of THSWD (Figures S7B-C, 5A).

**Figure 5:**
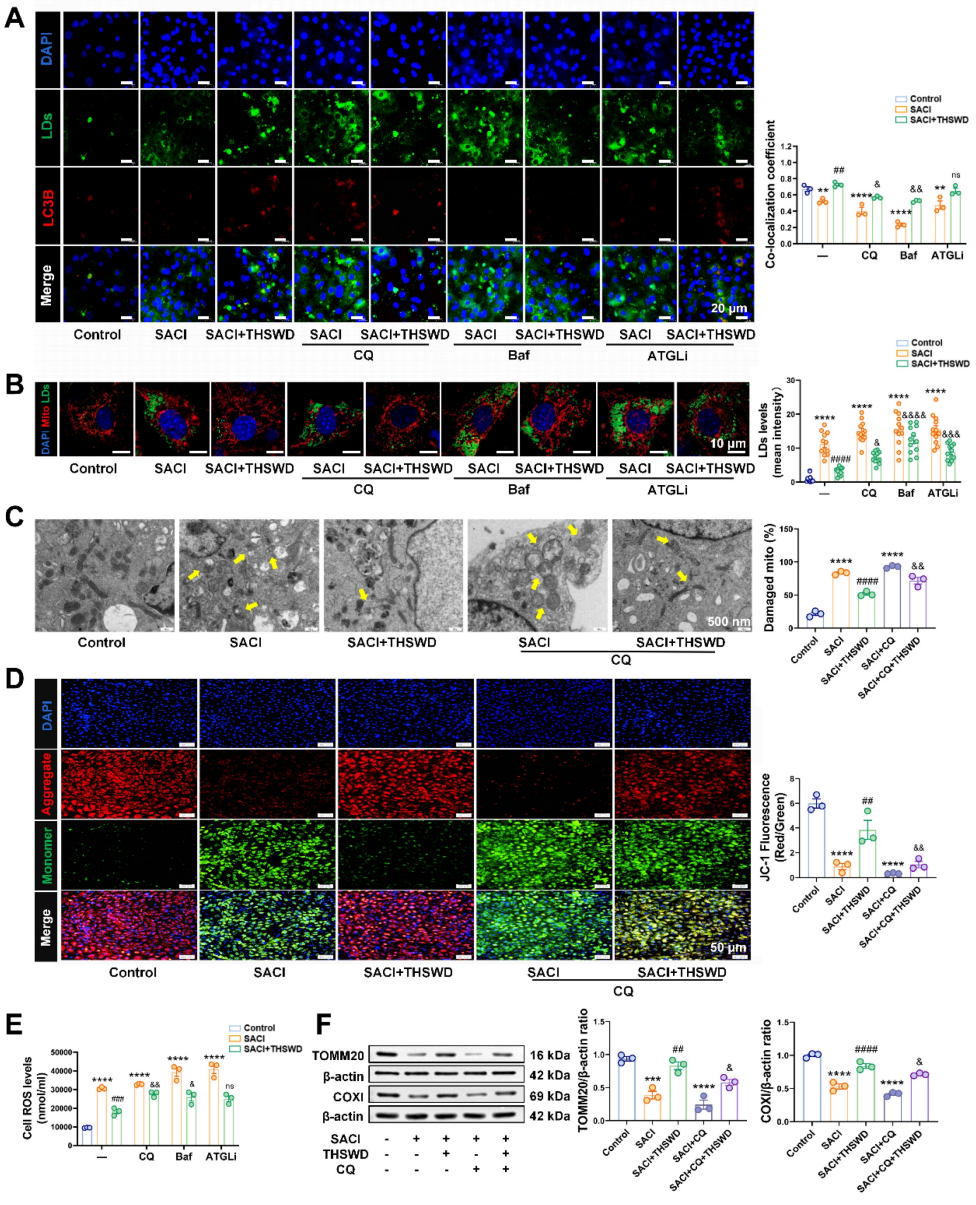
** THSWD attenuates lipotoxicity induced by SACI cardiomyocyte LDs by promoting lipophagy.** (**A**) Representative images of LC3B (red), LD (green), and nuclear (blue) signal and LC3B and LD signal colocalization in control, SACI, SACI+THSWD, SACI+CQ (100 μm, 1 h before SACI serum), SACI+CQ+THSWD, SACI+ATGLi (50 μM, 6 h with SACI serum), SACI+ATGLi+THSWD, SACI+Baf (100 nm, 1 h before SACI serum), and SACI+Baf+THSWD cultures (scale bar = 20 μm; n = 3/group). (**B**) Representative images of mitochondrial (red), LD (green) and nuclear (blue) signal and LD signal in these groups (scale bar = 10 μm; n = 12 cells/group). (**C**) Representative transmission electron microscopy images and damaged mitochondria percentages in these groups (scale bar = 500 nm; n = 3/group; arrows: damaged mitochondria). (**D**) Representative images and quantification of nuclear (blue) signal and JC-1 signal indicating normal (red) and decreased (green) mitochondrial membrane potential (ΔΨm) (scale bar = 50 μm; n = 3/group). (**E**) Intracellular ROS concentrations (n = 6/group) in these cultures. (**F**) Western blot analysis and quantitation of TOMM20 and COXI expression in these cultures (n = 3/group). Results are presented as mean ± SEM values. *p < 0.05, **p < 0.01, ***p < 0.005, ****p < 0.0001 vs. Control; #p < 0.05, ##p < 0.01, ####p < 0.0001 vs. SACI; &p < 0.05, &&p < 0.01, &&&p < 0.005, &&&&p < 0.0001 vs. SACI+THSWD. ns: nonsignificant vs. SACI+THSWD.

However, THSWD-induced lipophagy was not completely inhibited by ATGLi treatment to suppress lipolysis. CQ, Baf, and ATGLi all tended to increase LD accumulation and lipid peroxidation, [Ca^2+^]_i_ levels, and supernatant TNF-α and IL-6 concentrations consistent with increased SACI cardiomyocyte injury (Figures 5B, S7D). However, Baf and particularly ATGLi had much less consistent effects to inhibit the therapeutic effects of THSWD than CQ (Figures 5B, S7D). CQ treatment increased the severity of mitochondrial structural damage and attenuated a THSWD-mediated effect to attenuate this damage (Figure 5C). CQ treatment also increased mitochondrial and intracellular ROS production, and decreased TOMM20 and COXI expression and mitochondrial membrane potential in both SACI and SACI+THSWD cardiomyocyte cultures, and Baf and ATGLi effects on mitochondrial dysfunction were similar CQ effects (Figures 5D-F, S7E-F). Taken together, these results suggest that the THSWD effect to attenuate SACI cardiomyocyte lipotoxicity was primarily mediated by lipophagy rather than lipolysis.

### THSWD attenuates mitochondrial metabolic deficiencies of SACI cardiomyocytes

**Figure 6:**
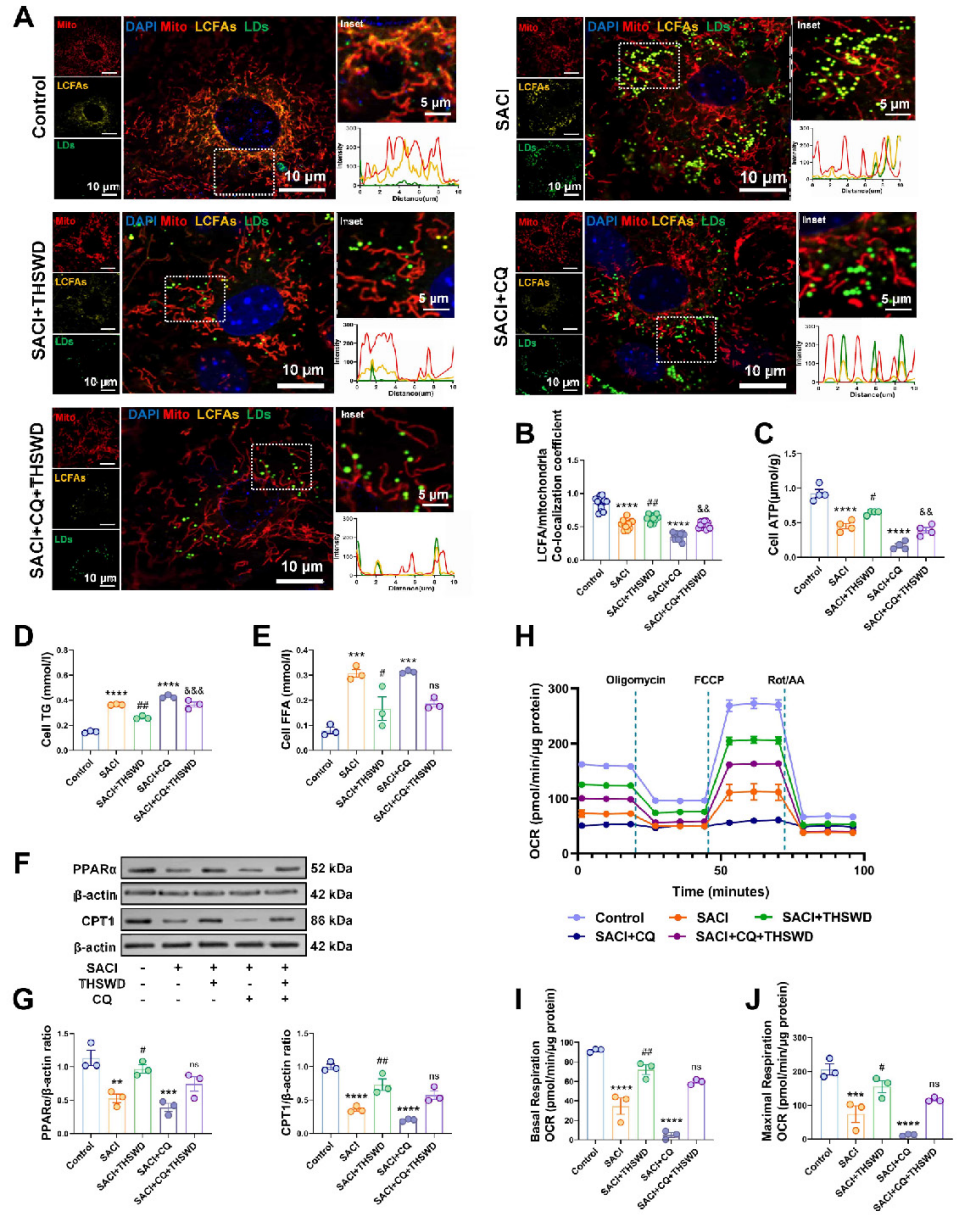
** THSWD attenuates mitochondrial metabolic deficiencies of SACI cardiomyocytes.** (**A**) Representative images and colocalization curves of mitochondrial (red), LCFA (gold), LD (green) and nuclear (blue) signals in control, SACI, SACI+THSWD, SACI+CQ, and SACI+CQ+THSWD cardiomyocyte cultures (scale bars = 5 μm and 10 μm; n = 12 cells/group). (**B**) LCFA/mitochondria colocalization coefficients (n = 12 cells/group), (**C**) ATP levels (n = 4/group), and (**D**) TG and (**E**) FFA concentrations (n = 3/group) in these culture groups. (**F**) Representative Western blot images and (**G**) graphs of PPAR-α and CPT1 expression and (**H**) the mean oxygen consumption rate (OCR), (**I**) basal respiration rate, and (**J**) maximal respiration capacity in these groups (n = 3/group). Results are presented as mean ± SEM values. ***p < 0.005, ****p < 0.0001 vs. Control; #p < 0.05, ##p < 0.01 vs. SACI; &&p < 0.01, &&&p < 0.005 vs. SACI+THSWD. ns: nonsignificant vs. SACI+THSWD.

Since LDs enriched in SACI cardiomyocytes store FAs employed as substrates in mitochondrial FA β-oxidation, we next analyzed the effects of THSWD on FA metabolism. SACI cardiomyocytes revealed substantial LD, TG, and FFA increases that were paired with reduced mitochondrial LCFA abundance, ATP production, and expression of the FA transporter CPT1 and its transcriptional regulator PPAR-α (Figures 6A-G), implying that the mitochondria of these cells could not efficiently use LD-derived LCFAs for energy production. THSWD treatment partially restored mitochondrial LCFA abundance and ATP production and reduced TG and FFA levels in these cells, whereas CQ treatment inhibited THSWD effects to restore mitochondrial FA metabolism and energy production and abrogate SACI-mediated increases in TG and FFA levels, indicating that increased lipophagy was at least partially responsible for these effects (Figures 6A-G). CQ treatment also partially attenuated THSWD-mediated restoration of mitochondrial oxidative metabolism (basal respiration, maximal respiratory capacity) in SACI cardiomyocytes (Figures 6H-J). These data thus strongly indicate that a THSWD-mediated effect to increase lipophagy can attenuate dysregulated LCFA transport to restore mitochondrial FA β-oxidation in SACI cardiomyocytes.

## Discussion

SAP often leads to myocardial injury and dysfunction, resulting in SACI 3. Consistent with previous studies 31, our SACI mice exhibited significant inflammatory responses, with elevated levels of lipase, amylase, proinflammatory cytokines (TNF-α and IL-6), cardiac injury markers (LDH, CK-MB, cTnI, and BNP), catecholamine secretion 32 and FFA levels, alongside cardiac tissue damage and abnormal LVEF and FS results. A previous study has reported that THSWD treatment can reduce RNA expression of several proinflammatory genes (TNF-α, MCP-1, and IL-1β) in the brain tissue of a rat middle cerebral artery occlusion model 33, similarly we observed that THSWD treatment effectively reduced the expression of proinflammatory factors and myocardial injury markers and mitigated inflammation and cardiac injury in our SACI mouse model. Network pharmacology also suggested that major THSWD components could influence TNF and HIF-1α signaling, lipid metabolism, and ATP synthesis, suggesting its potential utility as a therapeutic agent for conditions associated with the dysregulation of these pathways.

Normally, cardiomyocyte LDs stores excess lipids to prevent their damage to the heart and release TGs required to supply FFA substrates for mitochondrial β-oxidation and cellular energy metabolism 4, 34, but excessive LD accumulation leads to toxic TG, ceramides, glycerophospholipid, and sphingolipid increases that contribute to cardiac steatosis and dysfunction 35-37. Our proteomics analysis detected increased expression of the LD-associated protein Plin2, whose upregulation has been implicated in atrial sclerosis and atrial fibrillation 38, in the cardiac tissue of our SACI mouse model. These SACI mice also revealed elevated levels of lipid intermediates, ROS production 39, 40, and lipid peroxidation markers: MDA and 8-isoprostane 41, and calcium flux abnormalities 42 and inflammatory injury, all of which were consistent with lipotoxicity, and could be attenuated by Plin2 knockdown in SACI cardiomyocyte cultures. Notably, THSWD treatment was found to mimic most of therapeutic effects of Plin2 knockdown in SACI cardiomyocytes, as it significantly reduced Plin2 expression, accumulation of LDs and toxic levels of lipid intermediates 43, and ROS production and lipid peroxidation to mitigate lipotoxicity.

Sepsis-induced cardiomyopathy (SICM) is associated with increased lipolysis but reduced synthesis of long-chain acylcarnitines that transport FAs to the mitochondria, resulting in reduced mitochondrial FA β-oxidation and abnormal FFA and TG accumulation 44-46.

Similarly, SACI cardiomyocytes revealed reduced expression of two proteins involved in FA transport, CPT1 and PPAR-α, and attenuated acylcarnitine accumulation in their mitochondria, which was associated with LD accumulation and reduced mitochondrial FA β-oxidation to contribute to the mitochondrial dysfunction, increased ROS production, and reduced ATP generation of these cells 46, 47. Notably, our results also indicate that Plin2 can interact with two mitochondrial proteins, TOMM20 and COXI, suggesting that LD accumulation in SACI cardiomyocytes could promote LD-mitochondrial interactions and thereby exacerbate lipotoxicity due to the attenuation of a compensatory lipophagy mechanism in these cells. THSWD treatment attenuated mitochondrial structure defects detected in SACI mouse cardiac tissue, and improved mitochondrial membrane potential and ATP production, consistent with a LD to mitochondria redistribution of FAs 43, indicating that THSWD can attenuate mitochondrial energy metabolism deficits and structural damage associated with SACI.

Lipophagy plays a critical role in regulating lipid accumulation and metabolic homeostasis 9 by releasing FAs stored to serve as substrates for mitochondrial FA β-oxidation, which can attenuate the lipotoxic effects of excessive FA accumulation 10. SACI mice analyzed in this study exhibited evidence of reduced LD autophagy, as indicated by P62 increases and LC3B-LD colocalization decreases 48, 49. SACI cardiomyocyte treatment with CQ, an autophagy inhibitor 50, or Baf, a vacuolar-type H^+^-ATPase inhibitor that blocks autophagosome-lysosome fusion 51, exacerbated the LD accumulation, lipid peroxidation, and mitochondrial dysfunction phenotypes of these cells. CQ also disrupted mitochondrial FA β-oxidation in SACI cardiomyocytes, as indicated by reduced LCFA and mitochondria colocalization and decreased ATP production in these cells, indicating the importance of lipophagy in mitigating SACI-induced lipotoxicity.

THSWD-treated cardiomyocytes also revealed LC3BII-to-LC3BI ratio increases, reduced P62 expression, and enhanced LC3B-LD colocalization 43. THSWD treatment also reduced SACI cardiomyocyte LD accumulation and mitochondria structure defects, and normalized mitochondrial FA transport and ATP production in these cells, suggesting that THSWD promotes lipophagy to attenuate SACI cardiomyocyte lipotoxicity. Correspondingly, CQ-mediated inhibition of lipophagy largely negated THSWD therapeutic effects on these phenotypes, whereas Baf, which inhibits the fusion of autophagosomes with lysosomes, and ATGLi which inhibits lipolysis 52, did not completely counteract these THSWD effects, although both exacerbated SACI cardiomyocyte lipotoxicity and mitochondrial dysfunction phenotypes in the absence of THSWD treatment.

Our findings strongly indicate that a THSWD-mediated effect to enhance lipophagy alleviates SACI-induced lipotoxicity and mitochondrial dysfunction in our model system. However, future studies are needed to validate support these findings, and identify specific THSWD components responsible for its activity, and provide elucidate the precise molecular mechanism(s) through which THSWD regulates lipophagy. Several potential interactions we detected between Plin2 and major THSWD components and between Plin2 and TOMM20 and COXI are prime candidates for such studies.

In conclusion, our findings indicate that THSWD represents a promising therapeutic approach for SACI, since it can effectively increase lipophagy to attenuate SACI-associated myocardial lipotoxicity by attenuating excessive LD accumulation, which disrupts lipid metabolism and mitochondrial function in affected cardiomyocytes.

## Supplementary Material

Supplementary figures and tables.

## Abbreviations

ATGLi: Atglistatin
ATP: Adenosine Triphosphate
Baf: bafilomycin A1
CK-MB: creatine kinase MB isoenzyme
CPT1: carnitine palmitoyl-transferase 1
CQ: Chloroquine
cTnI: cardiac troponin I
FAO: fatty acid oxidation
FBS: fetal bovine serum
FFA: free fatty acids
FA: fatty acids
FS: fractional shortening
GO: gene ontology
IL-6: interleukin-6
KEGG: Kyoto Encyclopedia of Genes and Genomes
LCFA: long-chain fatty acids
LDH: lactate dehydrogenase
LPS: lipopolysaccharide
LVEF: left ventricular ejection fraction
MDA: malondialdehyde
MODS: multiple organ failure disorder
OPLS-DA: Orthogonal Partial Least Squares-Discriminant Analysis
PA: palmitate
PPAR-α: peroxisome proliferator-activated receptor-α
ROS: reactive oxygen species
SAP: Severe acute pancreatitis
SACI: severe acute pancreatitis-associated acute cardiac injury
SAR: selective autophagy receptor
SICM: sepsis-induced cardiomyopathy
LDs: Lipid droplets
TEM: transmission electron microscopy
TG: triglycerides
THSWD: Taohong Siwu decoction
TNF-α: tumor necrosis factor-α
UPLC-MS/MS: ultra performance liquid chromatography-mass spectrometry
