# Supplementary material for: Autophagy-driven lipid regulation by an herbal decoction alleviates cardiac lipotoxicity in severe acute pancreatitis
Source: Theranostics. 2025 Aug 11;15(17):8822–39. doi: 10.7150/thno.117243 (PMC12439263; doi:10.7150/thno.117243)
Supplement: Supplementary file 1 — Supplementary figures and tables. [file thnov15p8822s1.pdf]

Supplementary Information

Autophagy-driven lipid regulation by an herbal decoction alleviates cardiac lipotoxicity in severe acute pancreatitis

Yue Yang [a], Qian Hu [a], Hongxin Kang [a], Long Xie [b], Yue Hu [a], Juan Li [a], Xianlin Zhao [a], Lv Zhu [a], Wenfu Tang [a], Jingping Liu [c], Christopher J. Lyon[d, e], Meihua Wan [a, b, *]

a West China Center of Excellence for Pancreatitis, Institute of Integrated Traditional Chinese and Western Medicine, West China Hospital, Sichuan University, Chengdu 610041, China;

b The First People's Hospital of Shuangliu District, Chengdu 610299, China

c Department of Integrated Traditional Chinese and Western Medicine and NHC Key Laboratory of Transplant Engineering and Immunology, Frontiers Science Center for Disease-related Molecular Network, West China Hospital, Sichuan University, Chengdu, 610041, China

d Center of Cellular and Molecular Diagnosis, Tulane University School of Medicine, 1430 Tulane Ave. New Orleans, LA 70112, USA;

e Department of Biochemistry & Molecular Biology, Tulane University School of Medicine, 1430 Tulane Ave. New Orleans, LA 70112, USA.

* Corresponding author

Meihua Wan, West China Center of Excellence for Pancreatitis, Institute of Integrated Traditional Chinese and Western Medicine, West China Hospital, Sichuan University, Chengdu 610041, China; The First People's Hospital of Shuangliu District, Chengdu 610299, China

Email address: wanmh@scu.edu.cn (Meihua Wan)

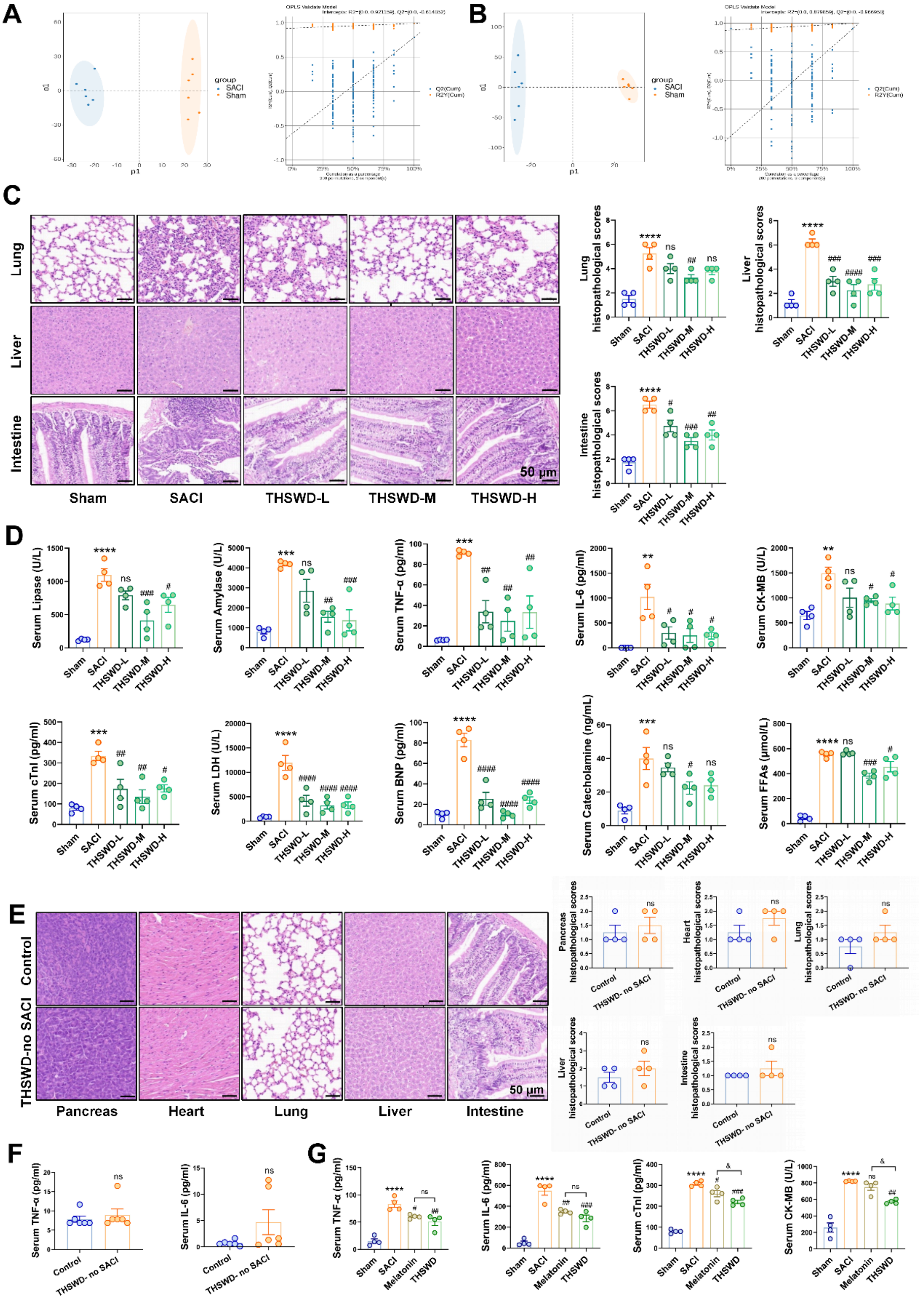

Figure S1. THSWD attenuates cardiac tissue changes related to SACI metabolic dysregulation. (A) Serum and (B) cardiac tissue orthogonal partial least squares discriminant analysis (OPLS-DA) model results for metabolomics data of the sham, SACI, SACI+THSWD groups (n = 6/group).(C) Representative images and histopathologic scoring of H&E stained lung, liver and intestine tissue specimens from uninjured (sham) and SACI mice treated with or without low, medium and high

THSWD doses (THSWD-L, THSWD-M, or THSWD-H) (scale bar = 50 µm; n = 4/group). (D) Serum lipase, amylase, TNF-α, IL-6, CK-MB, cTnI, LDH, BNP, catecholamine and FFA levels measured in these groups (n = 4/group). (E) Representative images and histopathologic scoring of H&E stained pancreas, heart, lung, liver and intestine tissue specimens in normal mice treated with (THSWD-no SACI) and without THSWD serum (control) (scale bar = 50 µm; n = 4/group). (F) Serum TNF-α and IL-6 levels detected in these groups (n = 6/group). (G) Serum TNF-α, IL-6, cTnI, and CK-MB levels measured in the sham, SACI, SACI+ melatonin, SACI+THSWD groups (n = 4/group). Results are presented as mean ± SEM values. *$p < 0.05$, **$p < 0.01$, ***$p < 0.005$, ****$p < 0.0001$ vs. sham. $^{\#\#}p < 0.01$, $^{\#\#\#}p < 0.005$, $^{\#\#\#\#}p < 0.0001$ vs. SACI. ns: nonsignificant vs. SACI.

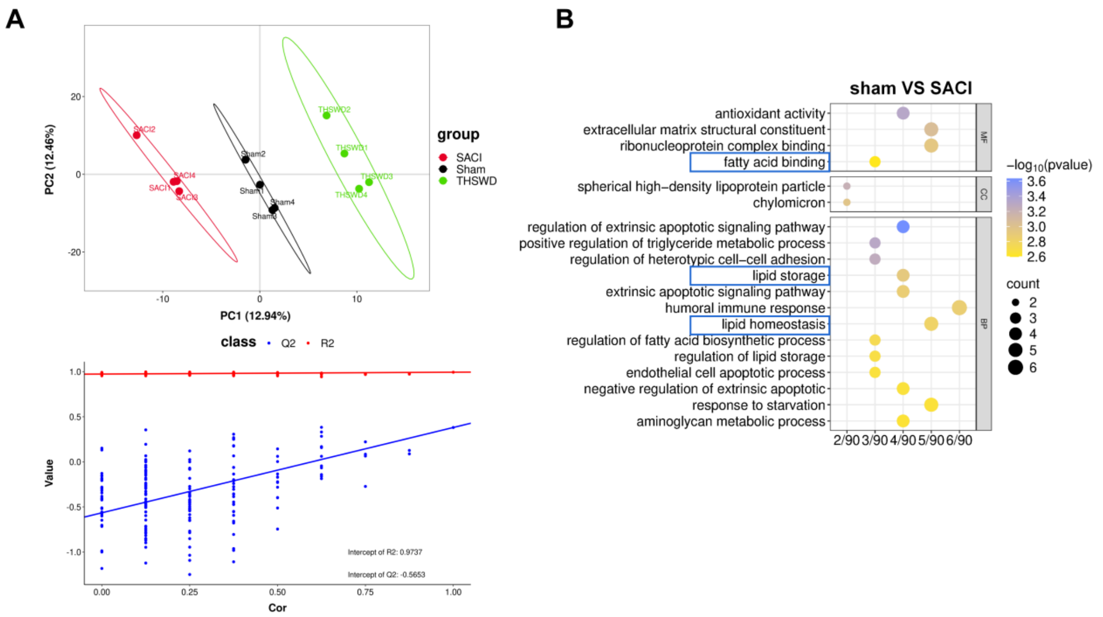

Figure S2. Bioinformatics analysis of Sham, SACI and SACI+THSWD mouse cardiac tissue proteomics data. (A) OPLS-DA model of cardiac tissue DEPs detected among the sham, SACI and SACI+ THSWD mouse groups (n = 4/group). (B) GO term analysis of DEPs in the cardiac tissues of sham vs. SACI mice (n = 4/group).

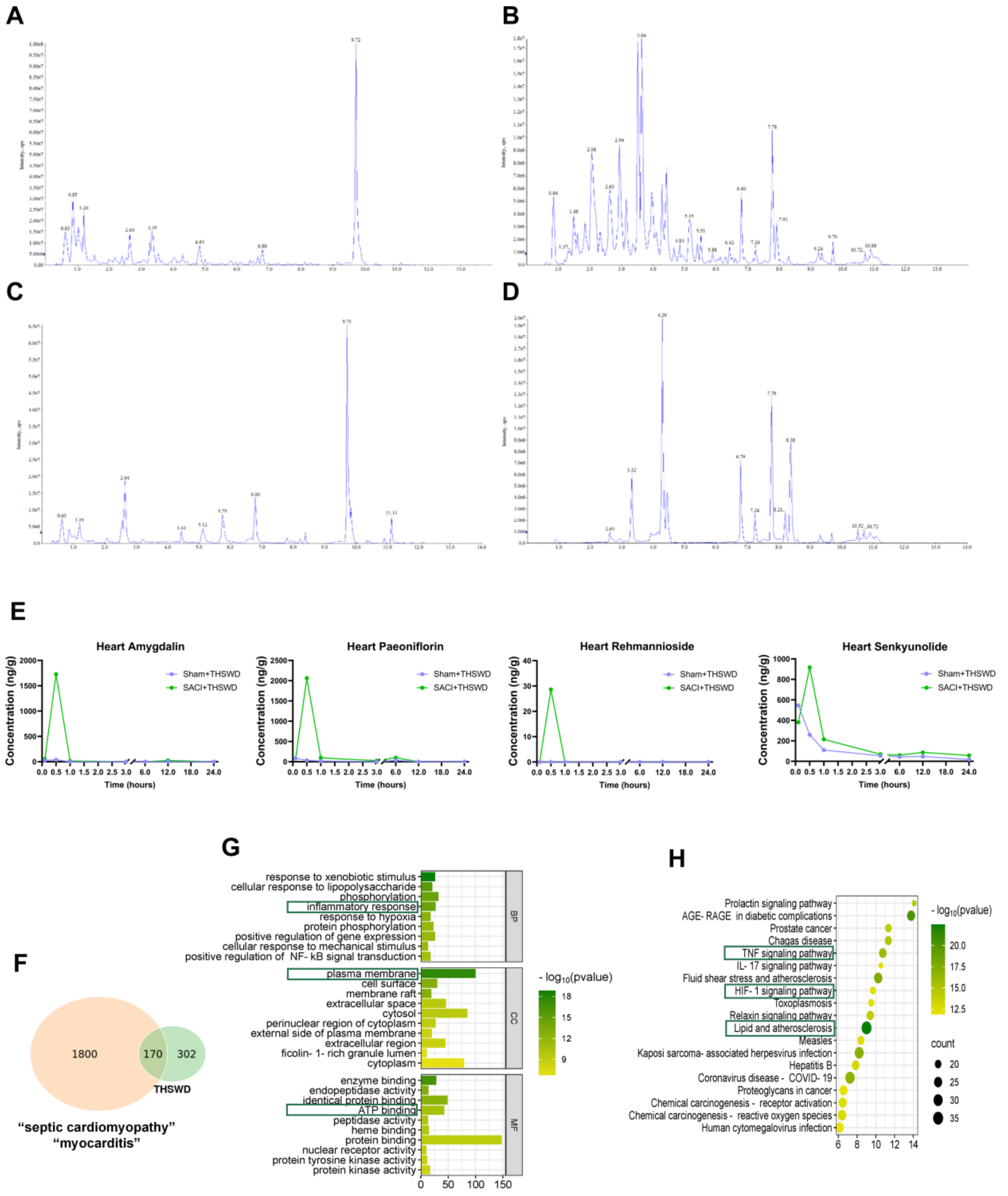

Figure S3. THSWD regulates cardiac tissue expression of genes associated with lipid storage and metabolism. (A-B) THSWD extract and (C-D) THSWD serum extract total ion chromatographs detected in positive (left) and negative (right) detection mode, respectively. (E) Cardiac tissue concentration-time profiles for six major THSWD components in Sham+THSWD and SACI+THSWD mice 10 and 30 min, and 1, 3, 6, 12, and 24 h after receiving of a medium THSWD dose. (F) Venn diagram of the overlap between (left) proteins associated with septic cardiomyopathy and myocarditis in a GeneCards search using these terms as keywords and (right) potential THSWD targets identified by screening the 16 THSWD-derived compounds detected in the serum against the PubChem and Swiss Target Prediction databases. (G) GO term enrichment analysis for these 170 shared protein targets, using the biological process (BP), molecular function (MF) and cellular component (CC) categories. (H) KEGG analysis of these 170 shared proteins.

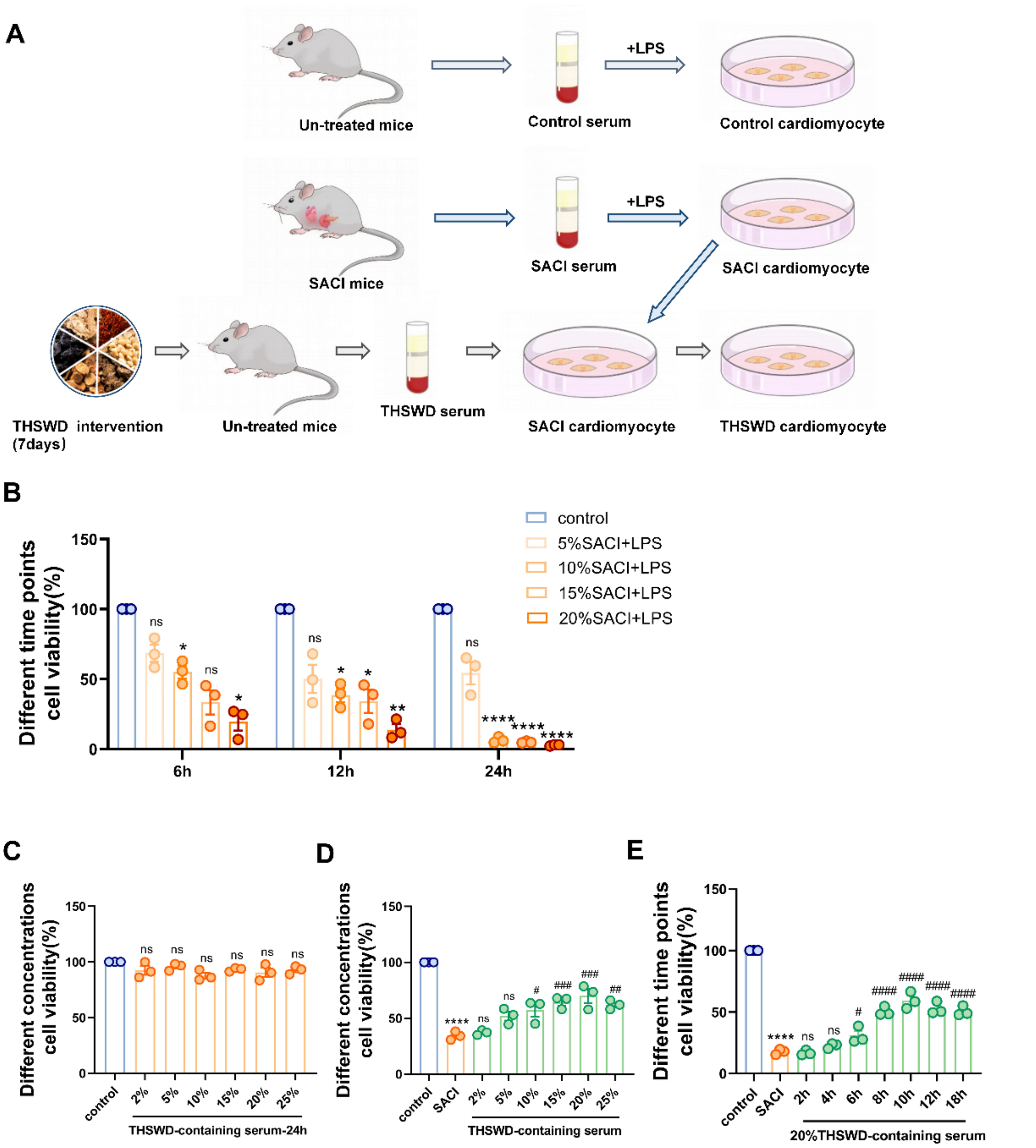

Figure S4. THSWD serum effects on sham control and SACI cardiomyocyte viability measured by CCK-8 assay. (A) Schematic of the cell culture protocol. (B) Cardiomyocyte survival after 6, 12, and 24 h exposure to the indicated concentrations of SACI serum and 1 μg/mL LPS (n = 3/group), where 6 h exposure to 10% SACI serum was selected to produce SACI cardiomyocyte. (C) Sham control and (D) SACI cardiomyocyte survival after 24 h exposure to the indicated THSWD serum concentrations (n = 3/group). (E) SACI cardiomyocyte survival after the indicated period of exposure to 20% THSWD serum (n = 3/group). Results are presented as mean ± SEM values. *$p < 0.05$, **$p < 0.01$, ****$p < 0.0001$ vs. the control; $^{\#}p < 0.05$, $^{\#\#}p < 0.01$, $^{\#\#\#}p < 0.005$, $^{\#\#\#\#}p < 0.0001$ vs. SACI. ns: nonsignificant vs. SACI.

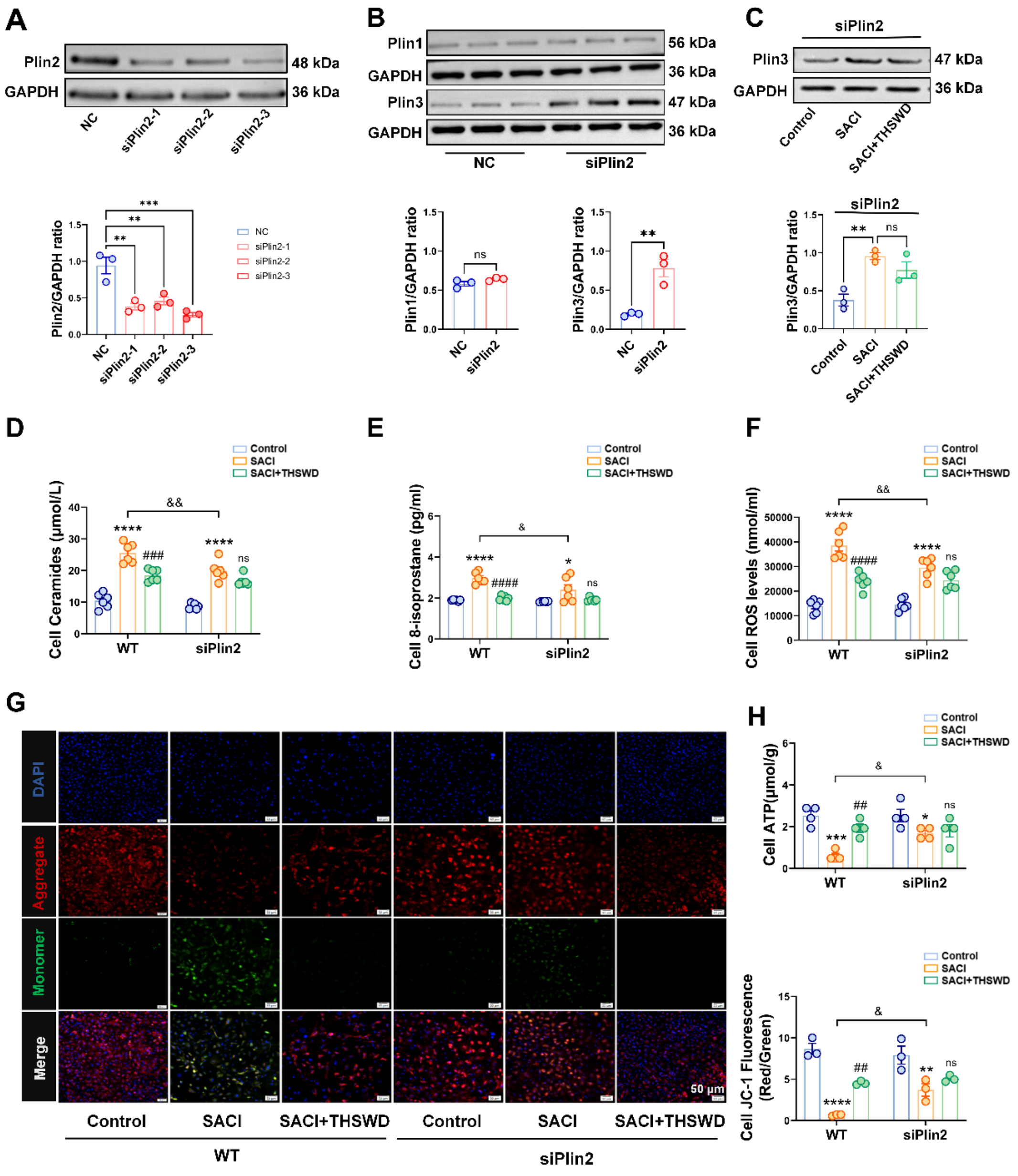

Figure S5. Plin2 knockdown mimics THSWD effects to attenuate SACI cardiomyocyte lipotoxicity. (A) Western blot and quantification of Plin2 knockdown in cardiomyocytes treated with the indicated Plin2-specific siRNAs (n = 3/group). (B) Western blot and quantification of Plin1 and Plin3 protein expression in the normal and siPlin2 cardiomyocyte cultures (n = 3/group). (C) Western blot and quantification of Plin3 protein expression in control, SACI, SACI+THSWD cardiomyocyte cultures after knockdown of Plin2 (n = 3/group). (D-E) Ceramides and 8-isoprostane levels measured in the supernatants of these cultures (n = 6/group). (F) Intracellular ROS concentration in these cultures (n = 6/group). (G) Representative images and quantitation of nuclear (blue) and JC-1 signal that indicates normal (red) and decreased (green) mitochondrial membrane potential (ΔΨm) (scale bar = 50 μm; n = 3/group), and (H) cardiomyocyte ATP levels in these cultures (n = 4/group). Results are presented as mean ± SEM values. $^{*}p < 0.05$, $^{**}p < 0.01$, $^{***}p < 0.005$, $^{****}p < 0.0001$ vs. Control; $^{\#\#}p < 0.01$, $^{\#\#\#}p < 0.005$, $^{\#\#\#\#}p < 0.0001$ vs. SACI; &p < 0.05, $^{\&\&}p < 0.01$ vs. WT SACI; ns: nonsignificant vs. siPlin2 SACI.

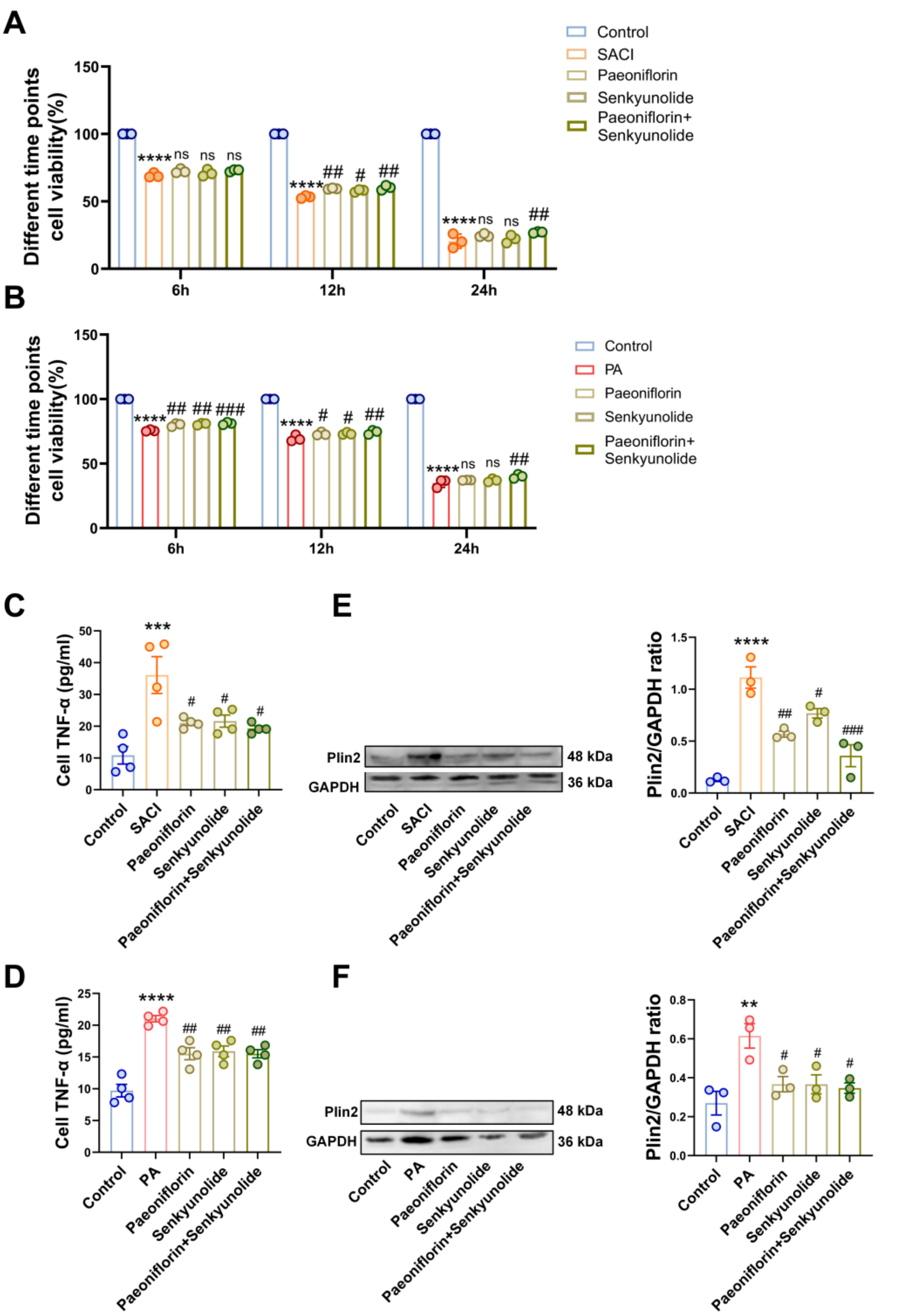

Figure S6. The effect of Paeoniflorin and Senkyunolide during continuous SACI serum and palmitate (PA) exposure. (A) Cardiomyocyte survival after 6, 12, and 24 hours of exposure to different intervention conditions (n = 3 cells/group), with control group: 10% control group (un-treated) mouse serum, SACI group: 10% SACI serum + LPS, SACI+ 100 μmol/L Paeoniflorin co-incubated group, SACI+ 1 mmol/L Senkyunolide co-incubated group and SACI+ 100 μmol/L Paeoniflorin+ 1 mmol/L Senkyunolide co-incubated group. (B) Cardiomyocyte survival after 6, 12, and 24 hours of exposure to different intervention conditions (n = 3 cells/group), with control group: 0.5% BSA group, PA group: 250 μ M palmitate/BSA solution, PA + 100 μ mol/L Paeoniflorin co-incubated group, PA + 1 mmol/L Senkyunolide co-incubated group and PA + 100 μ mol/L Paeoniflorin+ 1 mmol/L Senkyunolide co-incubated group. (C, D) TNF-α level measured in the supernatants after 12 h in SACI cardiomyocyte cultures, and after 6 h in PA

cardiomyocyte cultures (n = 4/group). (E, F) Western blot and quantification of Plin2 protein expression after 12 h in SACI cardiomyocyte cultures, and after 6 h in PA cardiomyocyte cultures (n = 3/group). Results are presented as mean ± SEM values. $^{*}p < 0.05$, $^{**}p < 0.01$, $^{***}p < 0.005$, $^{****}p < 0.0001$ vs. Control; $^{\#}p < 0.05$, $^{\#\#}p < 0.01$, $^{\#\#\#}p < 0.005$, $^{\#\#\#\#}p < 0.0001$ vs. SACI or PA; ns: nonsignificant vs. SACI or PA.

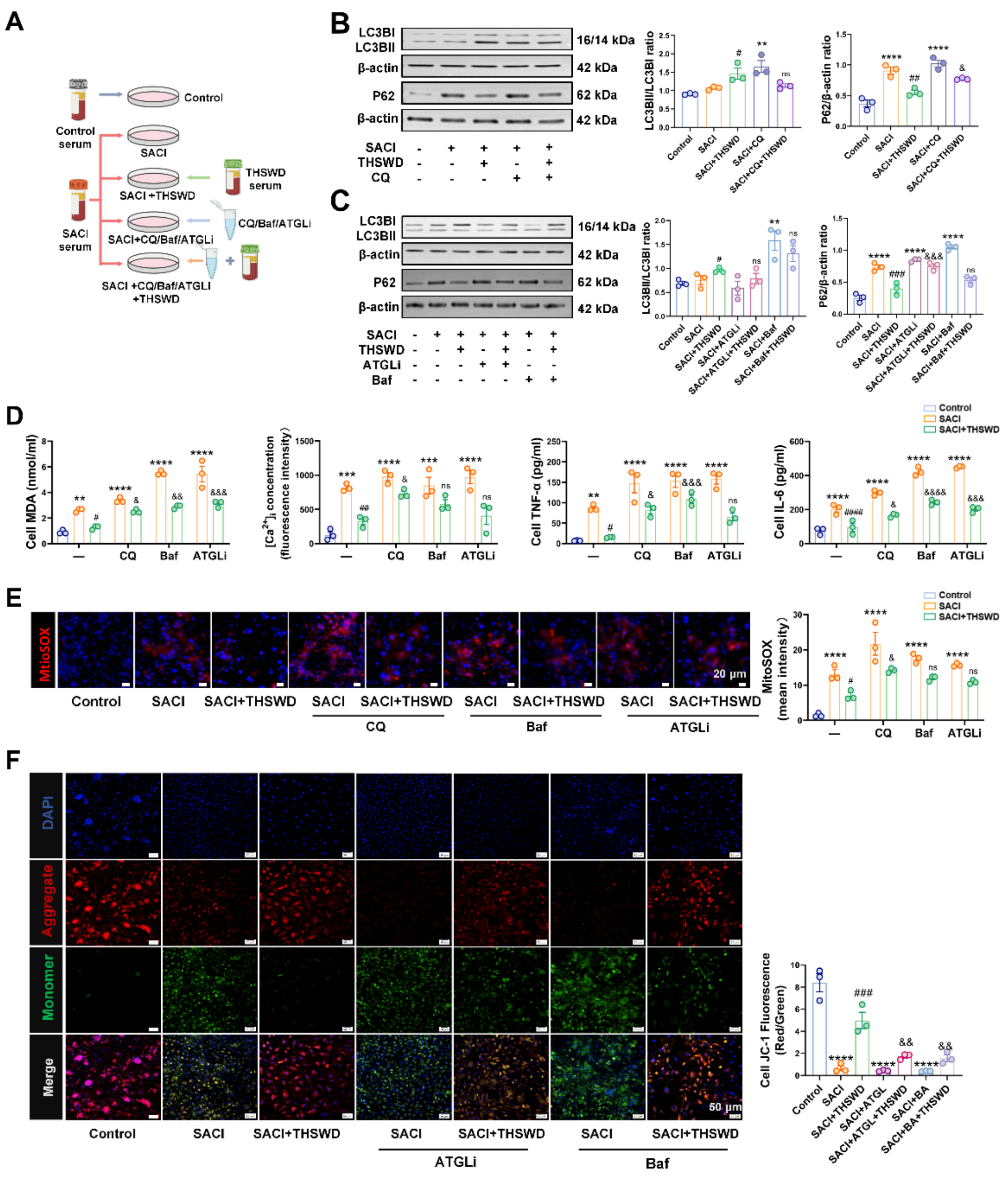

Figure S7. THSWD attenuates LD-induced cardiomyocyte lipotoxicity by promoting lipophagy. (A) Schematic of the cardiomyocyte treatment groups. (B) Western blot and quantification of LC3B and P62 expression in sham control, SACI, SACI+THSWD, SACI+CQ (100 μm, 1 h before SACI serum), and SACI+CQ+THSWD cultures (n = 3/group). (C) Western blot images and quantitation of LC3B and P62 expression in control, SACI, SACI+THSWD, SACI+ATGLi (50 μM, 6 h with SACI serum), SACI+ATGLi+THSWD, SACI+Baf (100 nm, 1 h before SACI serum), SACI+Baf+THSWD cultures (n = 3/group). (D) Supernatant MDA levels, intracellular calcium ion ($[Ca^{2+}]_i$) fluorescence intensity and supernatant TNF-α, IL-6 levels in these cultures (n = 3/group). (E-F) Representative

images and quantitation of (E) mitochondrial superoxide (MitoSOX) signal (red) (scale bar = 20 μm; n = 3/group) and (F) nuclear (blue) and JC-1 signal for normal (red) and decreased (green) mitochondrial membrane potential (ΔΨm) in these groups (scale bar = 50 μm; n = 3/group). Results are presented as mean ± SEM values. $^{**}p < 0.01$, $^{***}p < 0.005$, $^{****}p < 0.0001$ vs. Control; $^{\#}p < 0.05$, $^{\#\#}p < 0.01$, $^{\#\#\#}p < 0.005$, $^{\#\#\#\#}p < 0.0001$ vs. SACI; $^{\&}p < 0.05$, $^{\&\&}p < 0.01$, $^{\&\&\&}p < 0.005$, $^{\&\&\&\&}p < 0.0001$ vs. SACI+THSWD. ns: nonsignificant vs. SACI.

Table S1：RNAi sequences

si*Plin2 (ID: 11520)*	Sequence
Pair 1	CGGCUACGACGACACCGAUTT
	AUCGGUGUCGUCGUAGCCGTT
Pair 2	CCGCUUAUGUCAGUACAAATT
	UUUGUACUGACAUAAGCGGTT
Pair 3	GGACCAAGUCUGUGGUCAATT
	UUGACCACAGACUUGGUCCTT

Table S2: Major THSWD components detected by UPLC-MS/MS

	Identification	Molecular weight (Da)	Formula	CAS	THSWD prescription	THSWD-containing serum
1	Rehmannioside D	686.23	$C_{27}H_{42}O_{20}$	81720-08-3	1305828.45	0
2	Catalpol	362.12	$C_{15}H_{22}O_{10}$	2415-24-9	15015.67	604.07
3	Angelicin	186.03	$C_{11}H_6O_3$	523-50-2	1030663.81	31842.35
4	Ferulic acid	194.06	$C_{10}H_{10}O_4$	537-98-4	2480586.35	4413.51
5	Paeoniflorin	480.16	$C_{23}H_{28}O_{11}$	23180-57-6	43905372.31	58358.04
6	Paeonilactone B	196.07	$C_{10}H_{12}O_4$	98751-78-1	174809.41	20394.58
7	Paeonilactone C	318.11	$C_{17}H_{18}O_6$	98751-77-0	775683.27	1181.45
8	Albiflorin	480.16	$C_{23}H_{28}O_{11}$	39011-90-0	47891151.26	85756.58
9	8-Debenzoylpaeoniflorin	376.14	$C_{16}H_{24}O_{10}$	23532-11-8	100169.47	2502.46
10	Senkyunolide A	192.115	$C_{12}H_{16}O_2$	63038-10-8	435510.96	1026.91
11	Senkyunolide B	204.08	$C_{12}H_{12}O_3$	93236-67-0	916230.74	4238.86
12	Senkyunolide F	206.09	$C_{12}H_{14}O_3$	94530-84-4	693412.62	11855.76
13	Senkyunolide G	208.11	$C_{12}H_{16}O_3$	94530-85-5	1542103.62	3996.31
14	Senkyunolide H	224.10	$C_{12}H_{16}O_4$	94596-27-7	6582007.42	73472.58
15	Senkyunolide I	224.10	$C_{12}H_{16}O_4$	94596-28-8	174224.45	2857.70
16	Z-Ligustilide	190.10	$C_{12}H_{14}O_2$	4431-01-0	249784.81	787.33
17	Amygdalin	457.16	$C_{20}H_{27}NO_{11}$	29883-15-6	1740063.78	0
18	Picrocrocin	330.17	$C_{16}H_{26}O_7$	138-55-6	108067.40	5153.07
